# Anticancer Activities of the Quinone-Methide Triterpenes Maytenin and 22-β-hydroxymaytenin Obtained from Cultivated *Maytenus ilicifolia* Roots Associated with Down-Regulation of miRNA-27a and miR-20a/miR-17-5p

**DOI:** 10.3390/molecules25030760

**Published:** 2020-02-10

**Authors:** Camila Hernandes, Lucyene Miguita, Romario Oliveira de Sales, Elisangela de Paula Silva, Pedro Omori Ribeiro de Mendonça, Bruna Lorencini da Silva, Maria de Fatima Guarizo Klingbeil, Monica Beatriz Mathor, Erika Bevilaqua Rangel, Luciana Cavalheiro Marti, Juliana da Silva Coppede, Fabio Daumas Nunes, Ana Maria Soares Pereira, Patricia Severino

**Affiliations:** 1 Albert Einstein Research and Education Institute, Hospital Israelita Albert Einstein, São Paulo 05652-900, Brazil; camila.hernandes@einstein.br (C.H.); romariosallespva1@gmail.com (R.O.d.S.); elisahps92@gmail.com (E.d.P.S.); pedro.omori.mendonca@usp.br (P.O.R.d.M.); bruninha.lorencini@gmail.com (B.L.d.S.); erika.rangel@einstein.br (E.B.R.); luciana.marti@einstein.br (L.C.M.); 2 Department of Stomatology, School of Dentistry, University of São Paulo, São Paulo 05508-000, Brazil; lucyene_miguita@yahoo.com.br (L.M.); fadnunes@usp.br (F.D.N.); 3 Nuclear and Energy Research Institute IPEN-CNEN/SP, São Paulo 05508-000, Brazil; fakling@usp.br (M.d.F.G.K.); mathor@ipen.br (M.B.M.); 4 Unidade de Biotecnologia, Universidade de Ribeirão Preto, Ribeirão Preto 14096-900, Brazil; jujucoppede@gmail.com (J.d.S.C.); apereira@unaerp.br (A.M.S.P.)

**Keywords:** triterpene, squamous cell carcinoma, quinone-methide, microRNA

## Abstract

Natural triterpenes exhibit a wide range of biological activities. Since this group of secondary metabolites is structurally diverse, effects may vary due to distinct biochemical interactions within biological systems. In this work, we investigated the anticancer-related activities of the quinone-methide triterpene maytenin and its derivative compound 22-β-hydroxymaytenin, obtained from *Maytenus ilicifolia* roots cultivated in vitro. Their antiproliferative and pro-apoptotic activities were evaluated in monolayer and three-dimensional cultures of immortalized cell lines. Additionally, we investigated the toxicity of maytenin in SCID mice harboring tumors derived from a squamous cell carcinoma cell line. Both isolated molecules presented pronounced pro-apoptotic activities in four cell lines derived from head and neck squamous cell carcinomas, including a metastasis-derived cell line. The molecules also induced reactive oxygen species (ROS) and down-regulated microRNA-27a and microRNA-20a/miR-17-5p, corroborating with the literature data for triterpenoids. Intraperitoneal administration of maytenin to tumor-bearing mice did not lead to pronounced histopathological changes in kidney tissue, suggesting low nephrotoxicity. The wide-ranging activity of maytenin and 22-β-hydroxymaytenin in head and neck cancer cells indicates that these molecules should be further explored in plant biochemistry and biotechnology for therapeutic applications.

## 1. Introduction

Triterpenes, secondary metabolites ubiquitously distributed in the plant kingdom, comprise a large group of structurally diverse compounds. The most studied triterpenes are quinone-methides, a promising chemical class with innumerous biological activities, including anticancer effects [1,2,3]. Naturally occurring quinone-methide triterpenes (QMTs) can only be found as secondary metabolites in plants of the Celastraceae family and biotechnological techniques, such as in vitro culture of plant parts, currently provide enough material for research purposes [4]. Celastrol and its methyl ester, pristimerin, are the most studied QMTs. Originally extracted from the root bark of *T. wilfordii*, an ivy-like vine native to China, these compounds are now obtained from several other species. A comprehensive review on the antineoplastic potential of Celastraceae plant extracts and/or chemical constituents has been recently published [5].

In this study, the anticancer potential of two QMTs, maytenin and its derivative compound, 22-β-hydroxymaytenin, is reported. Maytenin and 22-β-hydroxymaytenin are secondary metabolites of *Maytenus ilicifolia*, a native plant from South America. The plant has been traditionally used for its anti-inflammatory, analgesic and antiulcerogenic properties since the 1930s; in 2012, it was formally added to the National List of Essential Medicines in the Brazilian Unified Health System (*Sistema Único de Saúde*—SUS) to be used as a phytomedicine. Antiproliferative and pro-apoptotic effects of the compounds obtained from *M. ilicifolia* roots cultivated in vitro were evaluated in cancer cell lines derived from head and neck squamous cell carcinoma (HNSCC). Since effects on monolayer cell culture can differ considerably from those obtained in a three-dimensional (3D) environment, we also assessed the effect of the compounds in spheroids derived from immortalized cell lines. Since microRNAs (miRNAs) have been implicated in the mechanisms of action of triterpenoids in association with the induction of ROS [6], we investigated the effects of maytenin and 22-β-hydroxymaytenin on the expression of miRNAs in normal oral keratinocytes and a HNSCC cell line using DNA microarrays.

HNSCC is responsible for about 90% of the cancers arising in the epithelial lining of the mucosal surfaces in head and neck [7]. Most patients are diagnosed at advanced cancer stages and over 50% of these patients will present recurrence in less than 2 years after initial treatment, with overall survival between 6 and 12 months [8]. Cisplatin [*cis*-diamminedichloroplatinum (II)] is an established and effective treatment for HNSCC, but even in combination with other agents such as 5-fluorouracil, the overall survival in recurrent or metastatic HNSCC is only around 10 months [9]. Cisplatin acts via crosslinking DNA, thus activating apoptosis in quickly dividing cells [10,11] but resistance involving a pleiotropic phenotype allows cells to resist cell death, a major challenge in cancer treatment [12,13,14]. Due to the established and effective bioactivity of cisplatin, antiproliferative and pro-apoptotic effects of the QMTs were compared to cisplatin treatments in vitro. An in vivo xenograft mouse model was used for the assessment of toxicity of maytenin through histopathological changes in kidney.

We propose a mechanism of action for the QMTs that include the down regulation of miRNAs in response to ROS, in agreement with previous reports for triterpenoids. This effect is not observed in similarly treated oral keratinocytes, or following treatment with cisplatin, despite the induction of ROS in these instances. Our results in vivo advocate that maytenin may be able to control cancer spread with a lower toxicity when compared with cisplatin, although further investigation is needed.

## 2. Results

### 2.1. Identification of Maytenin and 22-β-hydroxymaytenin in M. ilicifolia Root Extract 

**Figure 1:**
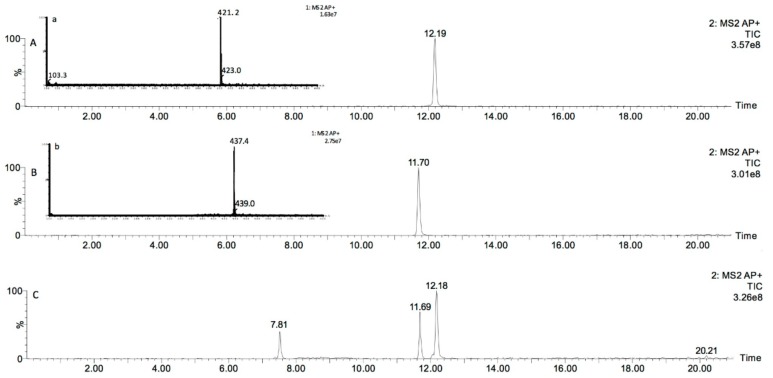
UPLC-MS chromatograms of maytenin (**A-a**), 22-β-hydroxymaytenin (**B-b**) and of dichloromethane roots extract from *M. ilicifolia* cultivated in vitro (**C**).

The raw dried extract obtained from the cultivated *M. ilicifolia* roots was analyzed by UPLC-DAD-MS (Figure 1A–C). Maytenin concentration in the raw extract was consistent, varying within 123.4 ± 3.1 µg/mL in each batch tested, and the proportion of 22-β-hydroxymaytenin was also reproducible.

### 2.2. QMTs Cytotoxic Effects in Squamous Cell Carcinoma Cell Lines and in Oral Keratinocytes 

**Figure 2:**
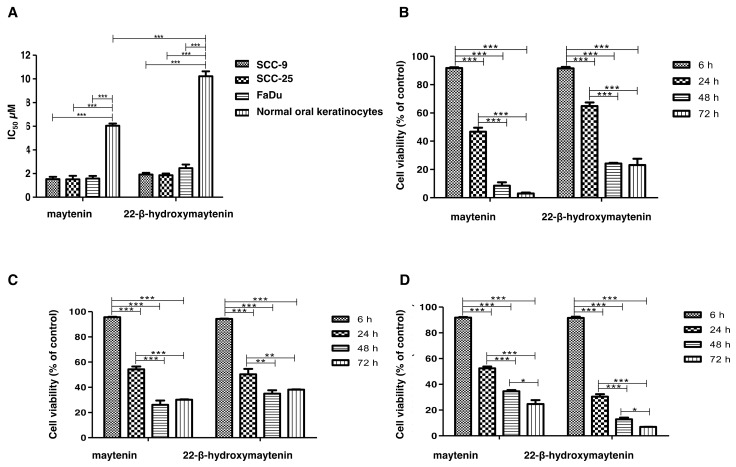
Cell viability results from MTT assay after incubation with maytenin or 22-β-hydroxymaytenin. (**A**) IC_50_ following 24 h incubation with maytenin or 22-β-hydroxymaytenin for each cell type; (**B**) Viability of SCC9 cell line following incubation for 6, 24, 48 or 72 h with maytenin or 22-β-hydroxymaytenin with IC_50_ value; (**C**) Viability of SCC25 cell line following incubation for 6, 24, 48 or 72 h with maytenin or 22-β-hydroxymaytenin with IC_50_ value; (**D**) Viability of FaDu cell line following incubation for 6, 24, 48 or 72 h with maytenin or 22-β-hydroxymaytenin with IC_50_ value. * *p* < 0.05, ** *p* < 0.01, *** *p* < 0.001 (one-way ANOVA).

**Figure 3:**
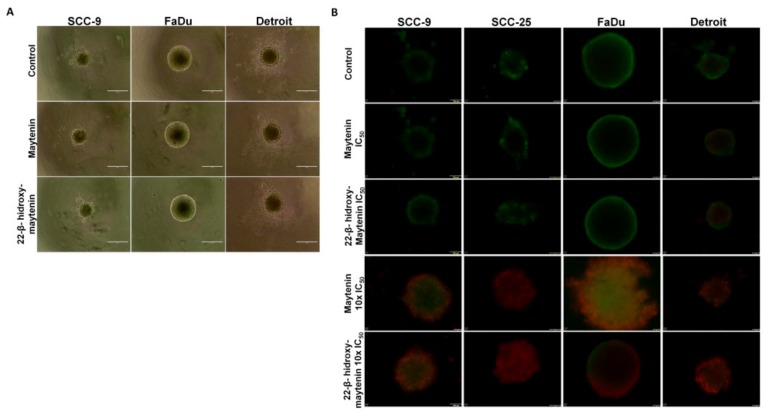
Size and cell viability in maytenin and 22-β-hydroxymaytenin treated spheroids. (**A**) Spheroids were treated for 48 h with the corresponding IC_50_ obtained in 2D cell culture. No size reduction was detectable using a light microscope for any of the treatments. *n* = 4. (**B**) Cell viability in the outer layers of the spheroids following treatment with the IC_50_ obtained in 2D cell culture and with 10× the IC_50_ obtained in 2D cell culture. *n* = 4. Live cells fluoresce green, and dead cells fluoresce red. A clear effect on cell viability was observed at the outer cell layer when 10× the IC_50_ was used for every treatment.

The effect of the QMTs on cell viability was determined by the MTT assay (Table 1). We observed that SCC cell lines responded similarly to the QMTs, while oral keratinocytes, used as a non-malignant cell model, were significantly less susceptible (Figure 2A). As shown in Figure 2 B-D, when SCC and FaDu cell lines were treated with IC_50_ values cell viability continued to decrease for 72h. Formal counting confirmed cell death (data not shown). When 3D cell cultures were treated with the same respective IC_50_ values from 2D cell culture, no effects in spheroid size or density were observed (Figure 3A), but cell death was confirmed in the outer layers when spheroids were treated with 10X the monolayer-determined IC_50_ value (Figure 3B). Despite cells growing as spheroids being more resistant to cell death than cells grown in monolayer, cell lines responded similarly to the treatments, highlighting a broad effect of the molecules in HNSCC-derived cell lines.

### 2.3. QMTs and Cisplatin Effects on Cell Proliferation

**Figure 4:**
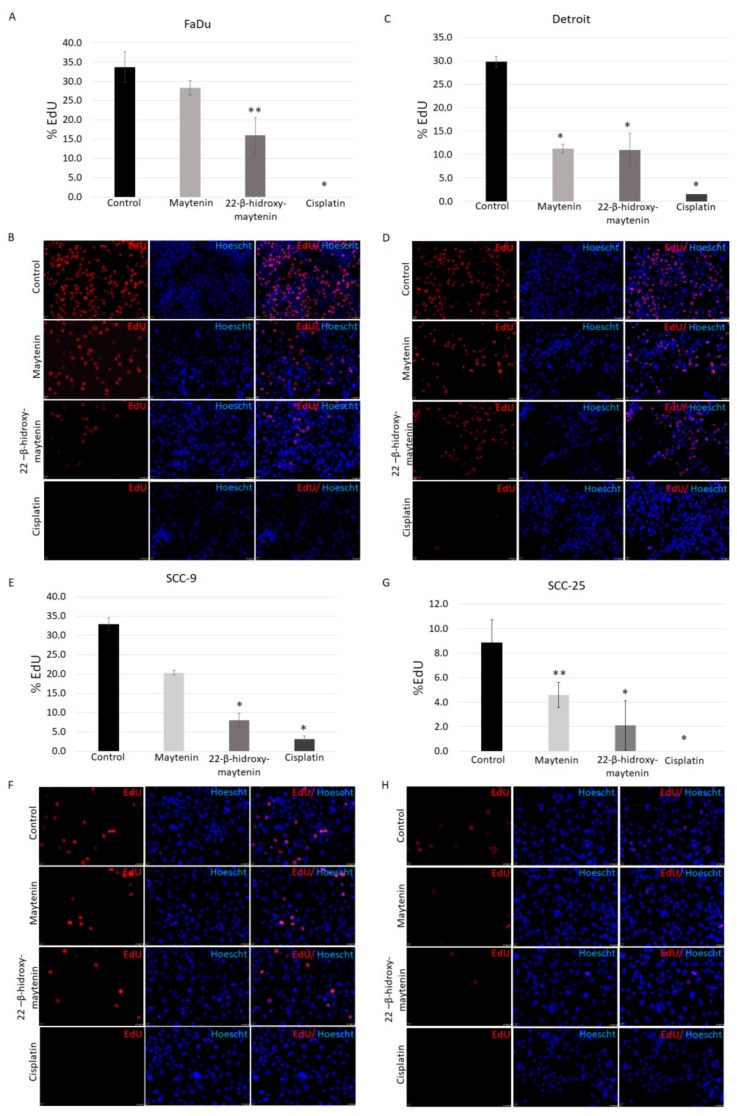
Anti-proliferative effects of maytenin, 22-β-hydroxymaytenin and cisplatin measured by EdU incorporation into DNA. Column graphs (**A**,**C**,**E**,**G**) show the percentage of cells staining for EdU (red) compared to Hoechst 33,342 (blue) stained nuclei (± SD). There were two replicates in each experiment and all cells were counted for each treatment. Images (**B**,**D**,**F**,**H**) were taken on a fluorescence microscope (magnification ×20) and depict EdU incorporation (red) and Hoechst 33,342 nuclei staining. EdU incorporation occurs during DNA synthesis, EdU positive cells indicate DNA replication and consequent cell cycle progression. * *p* < 0.01. ** *p* < 0.05.

The proliferative status of remaining cells following 24 h treatment with IC_50_ values was evaluated through the detection of DNA synthesis. While no DNA synthesis was detected in the remaining cisplatin-treated cells, QMTs treatment did not completely halt cell proliferation (Figure 4).

### 2.4. Oxidative Stress Induction and Apoptotic Cell Death via Caspase 3/7 Activation

**Figure 5:**
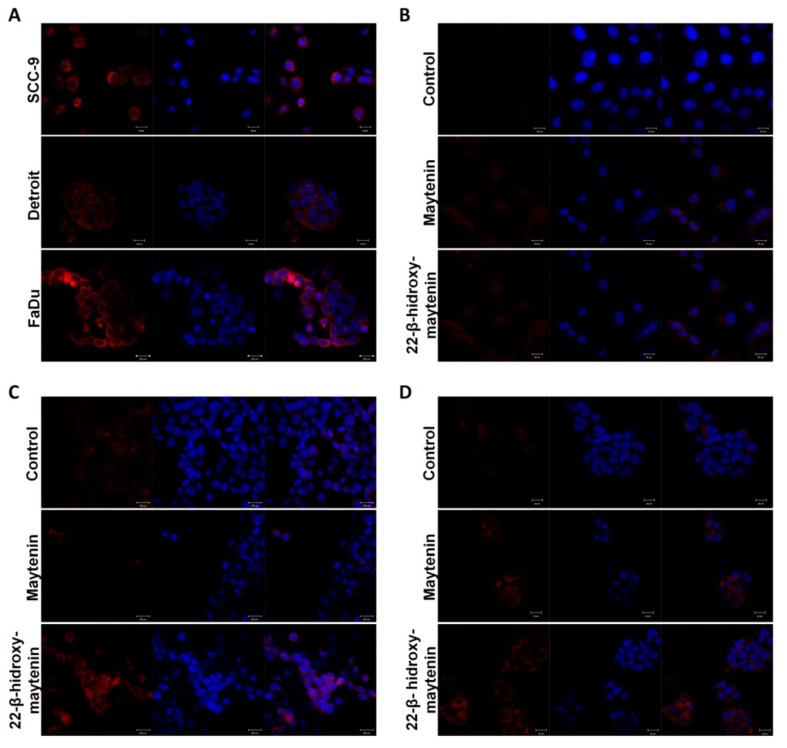
Effect of triterpenes on oxidative stress in SCC cell lines. (**A**) SCC9, Detroit and FaDu cell lines treated with cisplatin. ROS induction following cisplatin treatment is well established and these results were used as positive controls. The oxidative stress increases in tumor cells, compared with control when treated with maytenin or 22-β-hydroxymaytenin in SCC9 (**B**), FaDu (**C**) and Detroit (**D**). Total ROS after treatment with the IC_50_ value for 18 h was assessed by fluorescent probe detection using confocal microscopy. *n* = 2.

Cisplatin-induced generation of reactive oxygen species (ROS) has been correlated to its cytotoxic effects [15]. Detection of ROS was used to assess oxidative stress following 18 h treatment with QMTs. Since, after 24 h treatment, there was extensive cell death, we reasoned that induction of ROS should be seen prior to this result. Cisplatin was used as a positive control (Figure 5A). The qualitative results show higher levels of ROS following QMT treatment (Figure 5B,C).

**Figure 6:**
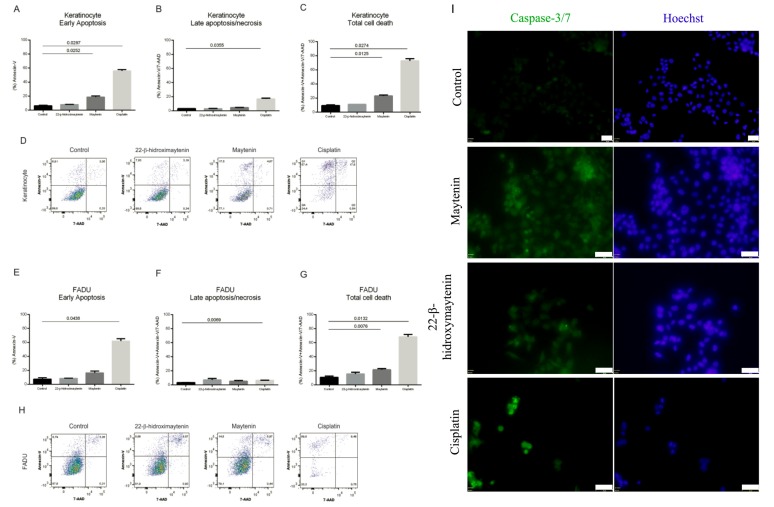
Apoptotic cell death measured by Annexin V and 7-ADD assay and caspase 3/7 activation. Keratinocytes and FaDu cell line were treated with 22-β-hydroxymaytenin, maytenin, or cisplatin and the effect on cell death induction was evaluated by flow cytometry using Annexin-V and 7-AAD staining. Keratinocytes: (**A**) Early apoptosis, (**B**) late apoptosis, (**C**) total cell death and (**D**) individual flow cytometry plots for each treatment. FaDu cell line: (**E**) Early apoptosis, (**F**) late apoptosis, (**G**) total cell death and (**H**) individual flow cytometry plots of each treatment. (**I**) caspase-3/7 activation in FaDu cell line following treatment by 22-β-hydroxymaytenin, maytenin, or cisplatin.

In order to further investigate cell death mechanisms, we looked for apoptotic changes in the plasma membrane using the annexin V assay. Figure 6 depicts the results for FaDu and normal keratinocytes. SCC9 and Detroit respond similarly (data not shown). Concurrent staining with Annexin-V and 7-AAD detected live cells, early stage of apoptosis and late stage of apoptosis following treatments. Total cell death was significantly distinct between treated (QMTs or cisplatin) and untreated cells (Figure 6A–H). The detection of caspase 3/7 activation following cell treatment confirmed programmed cell death proceeding via caspase 3/7 activation, both for keratinocytes and the cancer cell line (Figure 6I).

### 2.5. Maytenin and 22-β-hydroxymaytenin Intefere in miRNA Expression 

No broad range effect on miRNA gene expression was observed when cells were treated with maytenin, 22-β-hydroxymaytenin or cisplatin with a total of 145 and 69 miRNAs with some kind of differential regulation upon treament in oral keratinocytes and SCC25, respectively (Supplementary Materials Table S2A,B). Most regulated miRNAs in keratinocytes treated with QMTs were hsa-miR-3162-5p and hsa-miR-4530, both up regulated following treatment, but with no current function described. For the cell line, the two most regulated miRNAs were hsa-miR-21-5p and hsa-miR-205-5p, both down regulated. We looked specifically into the expression patterns of miR-27a and miR-20a/miR-17-5p, since they have been directly implicated in the mechanism of action of triterpenoids [6] (Table 2). Noteworthily, this group of miRNAs in SCC25 treated with maytenin and 22-β-hydroxymaytenin was down-regulated, as previously reported, but not when these cells were treated with cisplatin. A completely different result was found for treated keratinocytes, where no down regulation was observed (Table 2).

### 2.6. Effects of Maytenin on Metastasis and Renal Tissue Toxicity in Human Tumor Xenograft

**Figure 7:**
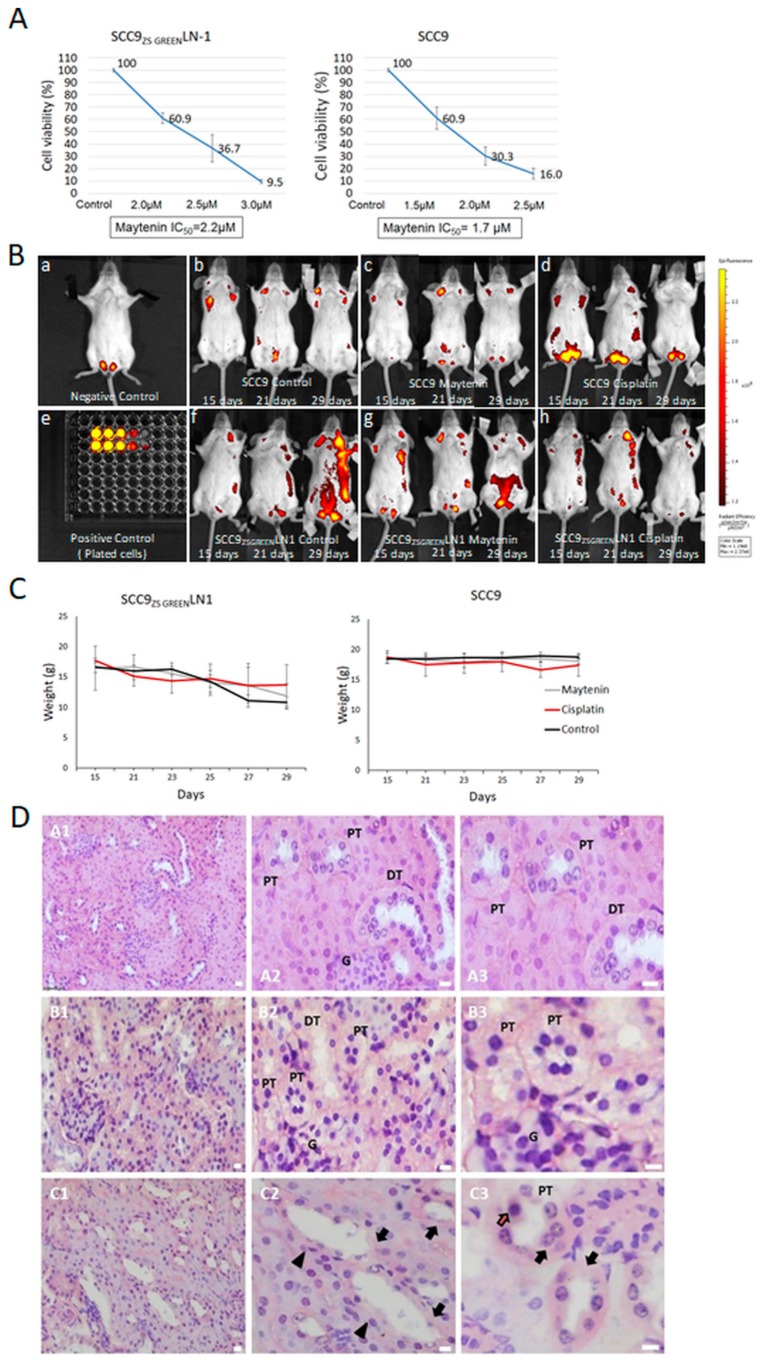
(**A**) Comparison of IC_50_ of maytenin for SCC9_ZS GREEN_ and SCC9_ZS GREEN_ LN-1. Maytenin showed similar effect in cell viability for SCC9_ZS GREEN_ and SCC9_ZS GREEN_ LN-1 cell lines. (**B**) Non-invasive in vivo imaging of mice harboring a human tumor xenograph prior and following treatment with maytenin and cisplatin: SCID mice were injected with SCC9_ZS GREEN_ or SCC9_ZS GREEN_ LN-1 cells and, 15 days later, mice with similar tumor burden determined by IVIS fluorescence imaging were assigned to each treatment group: control (PBS), maytenin (2 mg/Kg, i.p. Monday-Wednesday-Friday), or cisplatin (5 mg/Kg, i.p. once a week). b, c, d, f, g, h: representative IVIS images of mice from each experimental group at each stage the correspondent treatment: at 15 days following injection of cancer derived cells at the tongue mice had not received any treatment yet, at days 21 and 29, the respective treatment schemes were being applied. The graphical representation of the fluorescence in specific regions of interest in mice is indicated by a multi-color distribution: the high intensity of fluorescence appears as yellow color and low intensity of fluorescence as red. (**C**) Body weight of mice during the treatment cycle. There was no significant difference in body weight when maytenin and cisplatin were considered, but mice harbouring tumours derived from SCC9_ZS GREEN_ LN-1 cells lost weight due to tumour burden. (**D**) Photomicrograph of mouse kidney tissues with H&E staining: For untreated mice (A1, detailed in A2 and A3) and maytenin treated mice following injection of SCC9_ZS GREEN_ LN-1 cells at tongue (B1, detailed in B2 and B3) the cross section of kidney shows proximal tubules (PT) exhibiting brush border and distal tubules (DT) with normal morphology, the renal cortex exhibits normal glomerular (G) and tubular morphology structures. In cisplatin treated mice (C1, detailed in C2 and C3), renal cortex features suggest acute tubular necrosis as shown by patchy or diffuse denudation of the renal cells with loss of brush border (arrows), flattening of the renal tubular cells due to tubular dilation (arrowheads), pyknotic nucleus (red arrow) and loss of tubular architecture. Images illustrate the biological effects in tissue samples collected 29 days after the injection of SCC9_ZS GREEN_ LN-1 cells. Scale bars represent 20 µm.

Maytenin was tested in vivo aiming to assess its potential to control metastasis and to evaluate toxicity. SCID mice were injected at the submucosa of the tongue with the human oral cavity squamous cell carcinoma cell lines SCC9_ZS GREEN_ and SCC9_ZS GREEN_ LN-1. The cell lines SCC9_ZS GREEN_ and SCC9_ZS GREEN_ LN-1 stably express a green fluorescent zebrafish plasmid (ZsG) which allow us to follow their spread in vivo using and in vivo imaging of fluorescence system. The SCC9_ZS GREEN_ LN-1 cells are metastatic, derived from SCC-9 ZsGreen (refer to the Material and Methods section for further explanation). Beforehand, the dose responses of these modified cell lines were compared with the parental cell line SCC9 used in this work and showed similar results (Figure 7A). Mice were followed up for the presence of regional and other metastases using in vivo imaging of fluorescence and treatment started once metastases were detected. Cisplatin treatment scheme followed literature reports, and for maytenin, we chose a successful scheme presented for celastrol, another bioactive triterpene. After a 15 day treatment protocol, the chosen dose and treatment scheme for maytenin did not prevent cancer spread as efficiently as the cisplatin protocol (Figure 7B), but fewer metastases were seen in treated mice when compared with the untreated controls for the SCC9_ZS GREEN_ LN-1 cell line, a highly metastatic cell line (Figure 7B(f–h)). Due to the limited number of animals, no statistical result is presented for this analysis, but the observations indicate a potential effect in cancer progression for maytenin in vivo. We also report that the effect on body weight was similar for both maytenin and cisplatin (Figure 7C). Renal impairment is a major complication associated with cisplatin chemotherapy and histopathological alterations in this organ are well known. At the chosen dose, kidney sections from control and maytenin treated mice showed normal glomerular and tubular morphology structures in the renal cortex (Figure 7D(A1–A3),(B1–B3)). As expected, histopathological findings of the cisplatin treated mice showed degenerating tubular structures (Figure 7D(C1–C3)).

Hepatotoxicity associated with cisplatin chemotherapy is a rare event in clinical practice, but since maytenin and cisplatin are very different molecules, we accessed liver tissues in search for histopathological alterations possibly associated with treatment. Tumour cells infiltrating the organ, leading to architectural liver changes, were seen in untreated-mice, and, despite present, these events were fewer in cisplatin or maytenin treated-mice. One cisplatin-treated mouse exhibited focal haemorrhagic changes, which may suggest anoxia or toxic hepatopathies, and in maytenin-treated mice, we found areas of fibrosis, which could be a cytotoxic maytenin-mediated effect. These observations in treated mice are illustrated in Supplementary Figure S1 and may be associated with treatment, although more comprehensive animal studies must be proposed before a conclusion is reached.

## 3. Discussion

Treatment failure in HNSCC results primarily from regional and distant metastasis. Platinum-based chemoradiation is the standard treatment for patients with locoregionally advanced, recurrent and/or metastatic HNSCC. Possibly, the major development in first-line treatment for these cases in the past decade was the introduction of cetuximab—a monoclonal antibody which binds to the extracellular domain of the human epidermal growth factor receptor—in combination with platinum chemotherapy [16]. Despite the reported improvements in survival and life quality, the cost-effectiveness of the introduction of cetuximab in developing countries, including Brazil, is mostly unknown. In fact, cetuximab is not available for patients in the Brazilian Unified Health System (SUS) for the treatment of HNSCC. A recently published study addressing the cost-effectiveness of cetuximab for the treatment of colorectal cancer in Brazil points out that the antibody cannot be considered cost-effective from a SUS perspective with the registered prices [17]. In this scenario, cisplatin continues to be the most widely used standard agent in the treatment of HNSCC both in the locoregionally advanced and in the recurrent/metastatic disease settings in the country.

The need for novel therapeutic options is clear, but novel approaches need to undergo extensive preclinical studies before they may be considered remotely interesting for clinical trials. In this work, we performed a phenotypic analysis for *M. ilicifolia* QMTs maytenin and 22-β-hydroxymaytenin.

Two models for cell culture were used in this work, a monolayer cell culture (2D), and a three-dimensional model (spheroids). Dose-dependent experiments are traditionally performed in 2D cell culture in which the IC_50_ dose is determined. However, in the 2D cell culture, about 50% of the cell surface is dedicated to adherence to the flask, limiting cell–cell interactions and poorly representing the tridimensional structure of tissues [18]. Studies in the literature demonstrate that cells can behave differently in 3D models, becoming more resistant due to characteristics of cell interactions and extracellular matrix, and more proliferation is certainly seen at the outer layers. The IC_50_ for the two QMTs in monolayer cell culture was determined for four HNSCC-derived cell lines, each of them possessing distinct mutations associated with HNSCC development or progression. The equivalent results obtained here for the four cell lines suggest broad potential in targeting HNSCC. When tested in cells grown as spheroids, 10X the IC_50_ determined in the monolayer was needed for cell death in the outer layers, confirming the literature results for other compounds.

As determined by experiments using the incorporation of EdU into the DNA, indicating DNA synthesis, the QMTs did not halt cancer cells proliferation as efficiently as cisplatin, since live cells that remained in culture after treatment using IC_50_ values for QMTs were still proliferating, as opposed to cisplatin-treated cells. However, confirmed cell death both in monolayer cell culture, through the detection of caspase 3/7 activation, and in 3D cell culture, through ethidium homodimer-1 staining, indicate that the QMTs target cancer cells efficiently. Less oxidative stress, as measured by ROS detection, was also observed following QMT treatment of keratinocytes when compared with cell lines. Evidence has suggested that the involvement of oxidative stress in the pathogenesis of cisplatin nephrotoxicity [19,20], maytenin and 22-β-hydroxymaytenin may cause less harm in cancer free tissue due to less ROS induction.

Compounds studied as cancer chemopreventive agents, such as the antidiabetic drug metformin, curcumin, and also triterpenoid natural products, such as celastrol and its analogs [21,22,23] exhibit, besides exclusive activities, a common effect: they down regulate specific protein (Sp) transcription factors (TFs) Sp1, Sp3 and Sp4 and, consequently, pro-oncogenic Sp-regulated genes [24,25,26,27]. Some miRNAs over expressed in cancer, such as members of the miR-17-92 (miR-20a/miR-17-5p) and miR-27a clusters indirectly maintain high expression of Sp1, Sp3 and Sp4 [28,29]. It has been shown that triterpenoid-mediated induction of reactive oxygen species (ROS) is an important proteasome-independent pathway for down regulation of Sp transcription factors: ROS decreases expression of miR-27a and miR-20a/miR-17-5p, resulting in the induction of the Sp-repressors ZBTB10 and ZBTB4, respectively, which, in turn, down regulate Sp and Sp-regulated genes, such as Sp-regulated genes cyclin D1, bcl-2, survivin, PTTG-1 and EGFR [6,30,31]. Maytenin and 22-β-hydroxymaytenin clearly down-regulated miR-27a and miR-20a/miR-17-5p in the cancer cell line, but not in oral keratinocytes. Since fold-changes were expressed between treated and untreated cells, this result may simply indicate that in keratinocytes these miRNAs were not originally up regulated and therefore, suffered no clear effect from the treatment with QMT.

Noteworthily, the most down regulated miRNA following treatment with QMTs in the cancer cell line was miR-21-5p. This miRNA is overexpressed in many human malignancies and it has been shown to enhance chemoresistance to cisplatin [32,33,34]. The effect of QMTs on this miRNA could be explored as a therapeutic target.

Since maytenin and 22-β-hydroxymaytenin showed comparable results in vitro and maytenin is the major component in the root extract, it was chosen for an in vivo assay. In order to evaluate the toxicity of maytenin in vivo and to provide a preliminary result on the effect of maytenin on cancer progression, the QMT was tested following lingual injection of cells derived from a human squamous cell carcinoma of the tongue (a modified SCC9 expressing a green fluorescent zebrafish plasmid) in SCID mice (orthotopic xenoimplantation). Small-animal in vivo imaging based on bioluminescence or fluorescence is a viable solution to monitor xenotransplanted tumors in mice; it generates helpful results while diminishing the need for animal sacrifice [35]. With this technology and using an initial dose and treatment scheme based on previous reports for other triperpenes, we showed that maytenin may control cancer spread. Results with this QMT were inferior when compared with cisplatin, but it is important to note that treatment schemes using cisplatin are well established for mice, while here, we present a first proposal for maytenin. Additionally, cisplatin induces renal impairment and acute renal failure by induction of tubulointerstitial inflammation and apoptosis [19]. The search for strategies to prevent nephrotoxicity constitutes an active area of investigation [20]. We observed lower levels of nephrotoxicity in maytenin-treated mice. This lower toxicity indicates that higher doses may be considered and could lead to better results in preventing cancer progression. Noteworthily, cisplatin is known to cause significant histopathological changes in kidney tissue, therefore serving as a reference in this study. Even if similar changes had been observed, we could not suggest similar mechanisms of action since different molecular mechanisms may lead to similar tissue damage. Hepatotoxicity associated with cisplatin chemotherapy is a rare event in clinical practice; there are some data in rodents showing sinusoidal dilation and liver congestion [36] and dissolution of hepatic cords characterized by empty vacuoles aligned by strands of necrotic hepatocytes and inflammatory infiltrating cells, as well as periportal fibrosis [37]. Some architectural changes were observed in cisplatin-treated mice in this study, although a smaller amount when compared to untreated mice. In maytenin-treated mice, there were tumour cells infiltrating the organ associated with architectural liver changes, but also large areas of fibrosis. Those findings may be a cytotoxic maytenin-mediated effect but also a different behaviour of tumour growth after maytenin treatment. As reviewed by Oliveira C et al. [38], there are different patterns of tumour growth and it can have an impact on both prognosis and treatment. Thus, the tumour may permeate the liver without disrupting the normal architecture, or the tumour may be separated from the liver parenchyma by a band of fibrous tissue, which contains tumour-infiltrating lymphocytes, or the tumour may expand and compress the surrounding hepatocytes. In addition, fibrogenic changes were recently observed in patients treated with cisplatin/carboplatin/oxaliplatin-based regimens in colorectal and gastric cancer setting. Hepatic sinusoidal obstruction syndrome (SOS) was described in almost 40% of those cases and may adversely impact patient survival [39]. Further studies are required to address the hepatotoxicity associated with maytenin in dose and time-dependent manners, but this study provides a first histopathological view of both kidney and liver in mice following treatment with maytenin.

Compounds isolated from the Celastraceae family, which include *M. ilicifolia*, are continually investigated for antineoplastic effects. Studies address both plant extracts and its chemical constituents. QMTs are the most studied triterpenes from Celastraceae but this is the first report on anticancer effects of QMTs maytenin and 22-β-hydroxymaytenin. The production of these molecules is currently carried out using root cell cultivation under standardized conditions, and they constitute the most important bioactive elements of the raw extract. Taking into consideration the use of *M. ilicifolia* plant extract in current phytotherapy programs in the country, the knowledge on the bioactivity of its specific compounds may lead to increased efforts in research and novel applications.

## 4. Material and Methods

### 4.1. Maytenus Ilicifolia Root Cultivation In Vitro

A voucher specimen of *Maytenus ilicifolia* Mart. ex Reissek is deposited in the Herbarium of Medicinal Plants of the University of Ribeirao Preto. The authorization to use plant samples was given by Brazilian Institute of the Environment and Renewable Natural Resources (in Portuguese: *Instituto Brasileiro do Meio Ambiente e dos Recursos Naturais Renováveis*, acronym IBAMA), authorization number 010019/2015-4. The concentration of active molecules maytenin and 22-β-hydroxymaytenin in the source plant is typically low, approximately 0.0003%, and in order to produce enough material for research purposes, the in vitro cultivation of *M. ilicifolia* roots was optimized at the Plant Biotechnology Laboratory at Universidade de Ribeirao Preto [40]. Briefly, 1 cm of a *M. ilicifolia* plant were cultivated in vitro using Murashige and Skoog (MS) medium [41] supplemented with 30 g/L of sucrose and 2.5 g/L of Phytagel BioReagent (Sigma–Aldrich, St Louis, MO, USA) supplemented with 19.68 µM of indole butyric acid, 899.74 µM of polyvinylpyrrolidone and 30 g/L of glucose. After a 90 days period of explant inoculation, roots tissues were transferred to a liquid media containing 100 mL of Woody Plant Medium (WPM) [42] supplemented with 30 g/L of glucose, 19.68 µM of indole butyric acid and 899.74 µM of polyvinylpyrrolidone. Every 90 days, the medium was renewed. Liquid cultures were maintained in dark condition in agitation (90 rpm). One-year-old roots (1 g) were transferred to flasks containing 100 mL of WPM supplemented with 30 g/L of glucose, 19.68 µM of indole butyric acid and 899.74 µM of polyvinylpyrrolidone. After 21 days the roots were dried at 45 °C in a circulated air incubator for 48 h and then submitted to three consecutive extractions with dichloromethane for 30 min using ultrasound (Eco-Sonics). Once the solvent was removed by evaporation, the dried extract was analyzed by ultra-high-performance liquid chromatography–diode array detector–tandem mass spectrometry (UPLC-DAD-MS).

### 4.2. Instrumentation and Ultra-High-Performance Liquid Chromatography–Diode Array Detector–Tandem Mass Spectrometry (UPLC-DAD-MS) Analytical Conditions for Raw Extract Analysis 

In order to characterize the active compounds maytenin and 22-β-hydroxymaytenin present in the raw extract from cultivated *M. ilicifolia* root cells, 1 mg of the raw extract and authentic references for these compounds was solubilized in methanol to a final concentration of 50 µg/mL. All samples were filtered with 0.45 μm membrane syringe filters prior to analysis. A UPLC-MS analysis was performed using a Waters (Milford, MA, USA) Acquity UPLC H-Class system equipped with a PDA detector and a Waters Xevo TQ-S tandem quadrupole mass spectrometer with an atmospheric pressure chemical ionization (APCI) source operating in the positive mode. The samples injection volume was 5 µL in an Ascentis Express C18 column (100 × 4.6 mm i.d.; 2.7 µm particle size) from SUPELCO Analytical. The mobile phase used for gradient elution consisted of 0.1% formic acid (solvent A) and methanol containing 0.1% formic acid (solvent B) at a flow rate of 0.5 mL/min. The gradient elution program started with 30% B, raised B to 95% in the following 10 min, remained at 95% B for 5 min, and returned to the initial condition (30% B) within the following 5 min. The source and operating parameters were optimized as follows: corona voltage = 0.3 µA: APCI source temperature = 150 °C, desolvation temperature (N_2_) = 400 °C, desolvation gas flow = 250 l/h, and mass range from *m*/*z* 100 to 600 in the full-scan mode.

### 4.3. Quantification of Maytenin in the Raw Extract Using UPLC-MS/MS

For the quantification of maytenin in the raw extract using UPLC-MS/MS, authentic reference for this compound was diluted in methanol at 5, 10, 25, 50, 125, 250 and 500 ng/mL. The raw extract was precisely weighted (1.0 mg) and dissolved in 1 mL of methanol. The solution was filtered with 0.45 μm membrane syringe filters and a dilution to 10, 1, 0.1 e 0.01 µg/mL was performed. Ten μL of the raw extract was injected in an Ascentis Express C_18_ (100 × 4.6 mm, 2.7 µm) column by employing the Waters Acquity UPLC system. The mobile phase consisted of 0.1% formic acid aqueous solution (solvent A) and methanol supplemented with 0.1% formic acid (solvent B). A gradient elution started at 80% of solvent B and got to 95% during a 4-min period, stayed at 95% for 6 min and returned to 80% during the following 5 min. Optimal analysis conditions were determined and the calibration curve was used to quantify maytenin and 22-β-hydroxymaytenin. The concentration of the compounds in the raw extract is presented in μg/mL. The structures of maytenin and 22-β-hydroxymaytenin as determined by nuclear magnetic resonance (NMR) spectroscopy are presented as Supplementary Materials (Supplementary Files F1A and F1B).

### 4.4. Cell Culture

HNSCC-derived cell lines SCC9 (derived from a tongue squamous cell carcinoma, ATCC catalog number CRL-1629), SCC25 (derived from a tongue squamous cell carcinoma, ATCC catalog number CRL-1628), FaDu (derived from a hypopharynx squamous cell carcinoma, ATCC catalog number HBT43) and Detroit 562 (derived from a metastasis site, ATCC catalog number CCL-138). The main molecular characteristics of the ATCC cell lines can be found as supplementary material (Table S1). ATCC cell lines were purchased directly from *ATCC cell lines* (https://www.atcc.org) and even though cells are guaranteed to be free of mycoplasma by ATCC at the moment of the purchase, we routinely check for mycoplasma at our laboratory (EZ-PCR Mycoplasma test kit (Biological Industries^®^). For experiments in mice, SCC9_ZS GREEN_ and SCC9_ZS GREEN_ LN-1, kindly provided by Dr. Edgard Graner [43], were used. These cell lines stably express a green fluorescent zebrafish plasmid (ZsG) [44]. For the purpose of this study, the cell lines were grown in a Dulbecco’s Modified Eagle’s medium/Nutrient Mixture F-12 Ham (DMEM/F12) supplemented with 10% fetal bovine serum in a humidified atmosphere of 5% CO_2_ and 95% air at 37 °C. Human oral epithelial tissue was obtained from 3 healthy volunteers undergoing dental surgeries following approval by the Research Ethics Committee of *Instituto de Pesquisas Energéticas e Nucleares* (IPEN/CNEN-SP) (License Number 087/CEP-IPEN/SP) and informed consent signature. Keratinocytes derived from primary cultures of these cancer-free tissues were grown on a fibroblast (3T3-Swiss albino, ATCC catalog number CCL-92) feeder-layer in Dulbecco’s Modified Eagle Medium (DMEM; Gibco, New York, NY, USA) F-12 Nutrient Mixture (HAM, Gibco, New York, NY, USA) (2:1), with 10% Bovine Serum Product Fetal Clone III (Hyclone, Logan, Utah, USA), penicillin (100 U/mL), streptomycin (100 g/mL), gentamicin (50 g/mL), and amphotericin B (2.5 g/mL) glutamine (4 mM), adenine (0.18 mM) (Sigma–Aldrich, St. Louis, MO, USA), insulin (5 mg/mL) (Sigma–Aldrich, St Louis, MO, USA), hydrocortisone (0.4 g/mL) (Sigma–Aldrich, St Louis, MO, USA), cholera toxin (0.1 nM) (Sigma–Aldrich, St Louis, MO, USA), triiodotyronine (20 pM) (Sigma–Aldrich, St Louis, MO, USA) and epidermal growth factor (10 ng/mL) (R&D Systems, Minneapolis, MN, USA). Once the keratinocytes were confluent enough, cells are submitted to trypsin for dissociation (keratinocytes detach earlier than fibroblasts), the supernatant was collected and gently centrifuged. The pelleted keratinocytes were resuspended in culture medium, counted and plated in 96-well plates for the experiments. Experiments with cancer-free oral keratinocytes were performed with different batches in order to assess variability based on the original donor.

### 4.5. Determination of the Half-Maximal Inhibitory Concentration (IC50) for QMT: MTT Assay and Formal Counting 

The IC_50_ in the study was determined with the purpose of comparing the biological effects, i.e., proliferation inhibition, pro-apoptosis effect and ROS induction, of the two isolated QMTs. The colorimetric technique MTT (3-(4,5-dimethylthiazol-2-yl)-2,5-diphenyltetrazolium bromide) assay is currently the most extensively used method for IC_50_ measurements [45,46]. Cells were cultivated and treated with different concentrations in cell media of either maytenin, 22-β-hydroxymaytenin or cisplatin for 24 h in 96-well plates. Cell medium was removed and 100 µl of MTT Reagent (Sigma–Aldrich) in cell medium (0.5 mg/mL) was added to each well. Cells were incubated for 4 h, the medium was removed and 100 µL of DMSO (Sigma-Aldrich) was added to each well. Following incubation for 1 h at 37 °C, the absorbance in each well, including blanks wells containing medium only and wells containing untreated cells, was measured at 570 nm in a microtiter plate reader (Spectra max i3, Molecular Devices^®^). The average values were always determined from three independent wells and the average value for the blank was subtracted generating the final data. The IC 50 was estimated from a dose-response curve, correlating the percentage of viable cells and compound concentrations. Using Excel, compound concentrations and cell viability were plotted in a scatter plot (x and y axes respectively), a trend line was added and IC50 values (x) were then estimated using the equation of the line, when y = 0.5. The mean as well as the standard error obtained from each treatment in triplicate were calculated using the Graphpad Prism program (version 5.0). Due to reported MTT-dependent IC_50_ inconsistencies [45] formal counting was used to validate MTT data prior to biological assays. For formal counting, following the 24-h treatment, medium was removed and DNA staining was performed using Hoechst 33,342 (Thermofisher). Briefly, 1X Hoechst solution was added to each well (final concentration 5 μg/mL), cells were incubated for 30 min at room temperature, protected from light, and the Hoechst solution was then removed. Images were captured using an Olympus FSX-100 microscope equipped with the Olympus cellSens platform and cells were counted using the ImageJ software [47].

### 4.6. Analysis of In Vitro Cell Proliferation by EdU Incorporation

To label proliferating cells in vitro, we used the Click-iT^®^ EdU Alexa Fluor^®^ 594 Imaging Kit (Thermo Fisher Scientific, Walthan, MA, USA). This approach detects the synthesis of new DNA based on the incorporation of the nucleoside analog EdU (5-ethynyl-2’-deoxyuridine) into DNA. The fluorescent signal accumulates in the nuclei of cells where DNA has been synthesized during the EdU incubation period. Briefly, 10,000 cells were plated in 96-well plates and treated with maytenin, 22-β-hydroxymaytenin or cisplatin at IC_50_ for 24 h. There were at least two independent experiments for each cell line and in each experiment, cells were plated in triplicate. Cells were incubated with 1X EdU solution for 2 h after which the media was removed. The fixation step used 3.7% formaldehyde in PBS and it was followed by a 0.5% Triton^®^ X-100 in PBS permeabilization step. The Click-iT^®^ reaction cocktail was added to each well after the permeabilization buffer was removed. Proper incubation and washing steps followed the manufacturer’s protocol. For DNA staining using Hoechst 33,342 (Thermo Fisher Scientific, Walthan, MA, USA), each well was washed with PBS and 1X Hoechst 33,342 solution was added (final concentration 5 μg/mL). After an incubation period of 30 min at room temperature, protected from light, the Hoechst 33,342 solution was removed. Total cell count (Hoechst-positive cells) and proliferating cells (EdU-positive cells) were detected by fluorescence microscopy using the Olympus FSX-100 microscope equipped with the Olympus CellSens platform.

### 4.7. Cellular Oxidative Stress Assay

The CellROX^®^ Deep Red (Thermo Fisher Scientific, Walthan, MA, USA) was used to detect differential levels of oxidative stress following cell treatment. The reagent is non-fluorescent while in a reduced state it becomes fluorescent upon oxidation by ROS. The fluorescence is measurable by fluorescent imaging with emission maxima ~665 nm. Briefly, 10.000 cells were plated in specific slides (CellView Slide, Greiner) and treated with each compound at IC_50_ for 18 h. The CellROX^®^Reagent was added at a final concentration of 5 μM and cells were incubated for 30 min at 37 °C. Medium was removed, cells were washed 3 times with PBS and with 3.7% formaldehyde for 15 min. Hoechst 33,342 was used for nuclear counterstaining, following the protocol described in the previous section. Fluorescence was evaluated using a confocal microscope (Zeiss LSM 70 Observer Z1).

### 4.8. Detection of Apoptotic Changes by Annexin V Assay

The detection of apoptosis was carried out by concurrent staining with annexin V–FITC (Thermo Fisher Scientific, Walthan, MA, USA) and 7-aminoactinomycin D (7-AAD) (Beckman Coulter, Marseille, France) following treatment of cell lines and oral keratinocytes by QMTs. Cisplatin was used as a positive control due to its known proapoptotic effects. The Fluorescence Minus One Control (FMO) was used to correct spectral overlap and as a staining control. At least 5000 events were acquired for each condition using the flow cytometer FACSARIA II (BD Biosciences) and FlowJo software (TreeStar, Ashland, OR, USA) was used for data analyses. Early apoptosis was identified in the cells stained only with Annexin-V, late apoptosis or necrosis was identified in the cells stained with both Annexin-V and 7-AAD and unlabeled cells were considered viable.

### 4.9. Caspase-3/7 Activation Assay

Since cisplatin-induced programmed cell death has been shown to proceed via caspase-3 activation but alternative apoptotic pathways have been described [48], CellEvent™ Caspase-3/7 Green Detection Reagent was used for the detection of caspase-3/7 activation following cell treatment by QMTs. Cisplatin was used as a positive control. CellEvent Caspase-3/7 Green Detection Reagent is a four-amino acid peptide conjugated to a nucleic acid-binding dye with absorption/emission maxima of ~502/530 nm. After activation of caspase-3/7 in apoptotic cells, the peptide is cleaved, enabling the dye to bind to DNA and produce a fluorogenic response. The protocol consisted in adding the reagent to FaDu cells, incubating the cells for 30 min and visualizing the green nuclei of the apoptotic cells with activated caspase-3/7 and a minimal fluorescent signal in cells without activated caspase 3/7 (Olympus FSX-100 microscope equipped with the Olympus cellSens platform).

### 4.10. Formation of Spheroids with Cell Lines and Detection of Live and Dead Cells in their Outer Layers 

HNSCC cell lines (5000 cells/well) were cultured on Thermo Scientific™ Nunclon™ Sphera™ 96-well U-bottom plates for 7 days in complete medium. Spheroid formation was observed using light microscopy (Olympus FSX-100 microscope equipped with the Olympus cellSens platform). Spheroids were treated for 48 h with each QMT or with cisplatin. After 48 h, cell death was evaluated with the two-color LIVE/DEAD^®^ Viability/Cytotoxicity Kit (Thermo Fisher Scientific^®^). Briefly, a solution containing the green-fluorescent calcein-AM (stains live cells) and the red-fluorescent ethidium homodimer-1 (stains dead cells) was added to each well. Following 2 h of incubation with this solution images were analyzed using the FSX-100 microscope (Olympus^®^).

### 4.11. miRNA Expression Profiling

MiRNA expression profiling was undertaken for oral keratinocytes and SCC25 cell line using Human miRNA 8 × 60k Microarrays (SurePrint G4872A-046064, Agilent, Santa Clara, USA) following the manufacturer’s protocol. Cells were treated with cisplatin, maytenin or 22-β-hydroxymaytenin at IC_50_ for 24 h and compared with untreated cells. Data quality analyses were carried out using Feature Extraction Software following parameters recommended by the manufacturer (Agilent) and the GeneSpring software (Agilent) was used for data normalization and for the identification of differentially expressed genes. The results are shown in fold-change relative to untreated cells.

### 4.12. Animal Experiments for Kidney Toxicity Analysis following Cisplatin and Maytenin Treatment

The animal experiment was conducted in compliance with federal laws and with internationally accepted principles for laboratory animal use, and in strict accordance with the Committee for Ethics in Animal Research of *Instituto de Pesquisas Energéticas e Nucleares* (IPEN) policies and guidelines, São Paulo, Brazil (Approval number 201/17). SCC9 ZsGreen and SCC-9 ZsGreen LN-1 cells were grown until 60% to 70% confluence, and 10^5^ cells/20 μL of PBS were injected with a fine needle into the right lateral portion of the tongue of 4-week-old female SCID mice as previously described [49]. Before the procedure the animals were anesthetized with intraperitoneal injections of ketamine (100 mg/kg) and xylazine (10 mg/kg). Animals were divided in 3 groups of 4 animals: group 1—control (treatment with PBS), group 2—maytenin treatment and group 3—cisplatin treatment. Careful intraperitoneal (IP) injection of cisplatin and maytenin followed treatment schemes suggested in the literature, with the maytenin dose being calculated based on reports for other triterpenes [50,51]. We used an in vivo imaging system (IVIS Spectrum In Vivo Imaging System, PerkinElmer) to capture fluorescence and progressively track the invasion and dispersion of SCC9 ZsGreen and SCC-9 ZsGreen LN-1 cells in the mouse body. Total fluorescence was analyzed using the Live Image software (PerkinElmer). Fluorescence intensity within specific regions of individual animals was quantified using the region of the interest tools in the Live Image software. The equipment setting, image acquisitions and analysis followed the protocols recommended by the manufacturer. Briefly, treatment started once tumor dissemination was detected and from this moment on, IP administration of cisplatin (5 mg/Kg) was carried out once a week and maytenin (2 mg/Kg) three times per week. After 3 weeks (1 image pre-treatment: 15 days post the injection of cells; and two images during treatment: 21 days and 29 days after the injection of cells), the animals were sacrificed by overdose of anesthetics (2 times the anesthetic dose), and kidneys and liver were collected. Tissue samples were fixed in 10% formalin for immunohistochemistry.

## Figures and Tables

**Table 1:** IC_50_ following 24 h incubation for maytenin, 22-β-hydrodymaytenin and cisplatin as determined by MTT assay.

**IC_50_ (µM)**			
**Cell Types**	**Maytenin**	**22-β-hydrodymaytenin**	**Cisplatin**
SCC-9	1.5 ± 0.2	1.9 ± 0.2	59.1 ± 9.1
SCC-25	1.5 ± 0.3	1.9 ± 0.1	21 ± 3
FaDu	1.6 ± 0.2	2.5 ± 0.3	33.5 ± 0.6
keratinocytes	6 ± 0.2	10.2 ± 0.4	85.6 ± 2.1

**Table 2:** Fold changes in miR-27a and miR-20a/miR-17-5p expression in treated cells. Cells were treated with cisplatin, maytenin or 22-β-hydroxymaytenin at IC_50_ for 24 h and compared with untreated cells. miRBAse: http://www.mirbase.org/.

**Systematic miRNA Name**	**FC (SCC25 22b vs. SCC25 Untreated)**	**FC (SCC25 Maytenin vs. SCC25 Untreated)**	**FC (SCC25 Cisplatin vs. SCC25 Untreated)**	**miRBase Accession**
miR-17-5p	−36.4	−36.4	−1.3	MIMAT0000070
miR-20a-5p	−88.1	−88.1	−1.1	MIMAT0000075
miR-27a-3p	−62.0	−62.0	−1.2	MIMAT0000084
**Systematic miRNA Name**	**FC (Keratinocyte 22b vs. Keratinocyte Untreated)**	**FC (Keratinocyte Maytenin vs. Keratinocyte Untreated)**	**FC (Keratinocyte Cisplatin vs. Keratinocyte Untreated)**	**miRBase Accession**
miR-17-5p	−1.0	55.4	−1.0	MIMAT0000070
miR-20a-5p	1.7	8.3	3.4	MIMAT0000075
miR-27a-3p	1.9	7.9	2.9	MIMAT0000084

## Supplementary Materials

The Supplementary Materials are available online at https://www.mdpi.com/1420-3049/25/3/760/s1.
