# Supplementary material for: Anticancer Activities of the Quinone-Methide Triterpenes Maytenin and 22-β-hydroxymaytenin Obtained from Cultivated Maytenus ilicifolia Roots Associated with Down-Regulation of miRNA-27a and miR-20a/miR-17-5p
Source: Molecules. 2020 Feb 10;25(3):760. doi: 10.3390/molecules25030760 (PMC7038027; doi:10.3390/molecules25030760)
Supplement: Supplementary file 1 [file molecules-25-00760-s001.zip › Supplementary Figure 1.pdf]

Supplementary Figure 1: Histopathological alterations in liver

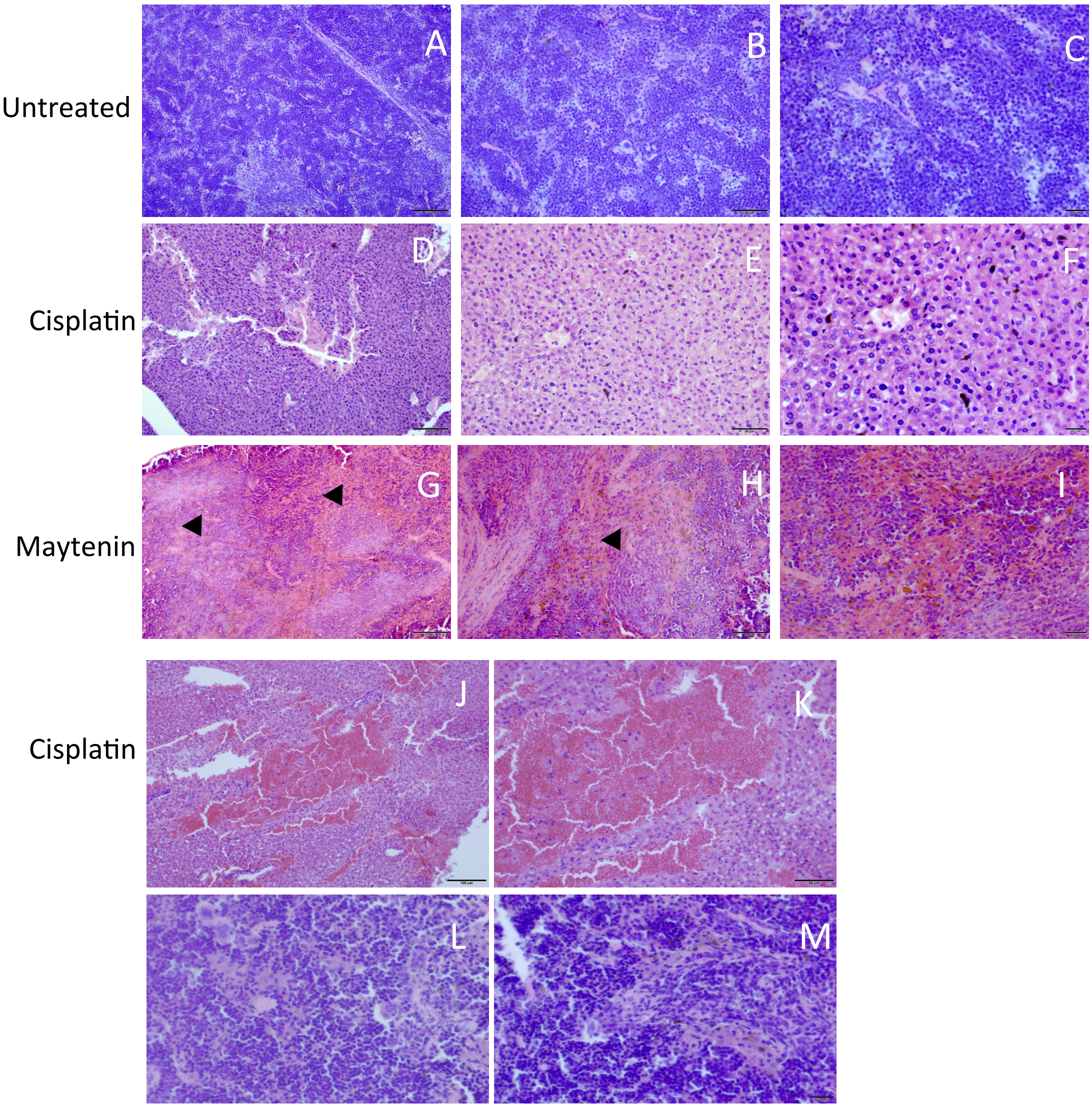

We found a large amount of tumour cells infiltrating the hepatocytes associated with architectural liver changes in untreated-mice (untreated: Figures 1 A-C), as opposed to cisplatin treated-mice (cisplatin: Figures 1 D-E). In maytenin-treated mice, we saw tumour cells infiltrating the hepatocytes associated with architectural liver changes, but also large areas of fibrosis (Figures 1 G-I), with some nodules surrounded by fibrous tissue (arrowheads, Figures 1 G-H). Additionally, in one cisplatin-treated mouse we identified focal haemorrhagic changes associated with vacuole degeneration (Figures 1 J-K), which may suggest anoxia or toxic hepathopathies. In another cisplatin-treated mouse, tumour cells infiltrating the hepatocytes and architectural changes were observed (Figures 1 L-M), although in less amount when compared to untreated-mice.
