# Supplementary material for: Anticancer Activities of the Quinone-Methide Triterpenes Maytenin and 22-β-hydroxymaytenin Obtained from Cultivated Maytenus ilicifolia Roots Associated with Down-Regulation of miRNA-27a and miR-20a/miR-17-5p
Source: Molecules. 2020 Feb 10;25(3):760. doi: 10.3390/molecules25030760 (PMC7038027; doi:10.3390/molecules25030760)
Supplement: Supplementary file 1 [file molecules-25-00760-s001.zip › Supplementary File F1 Molecules.pdf]

A: Maytenin

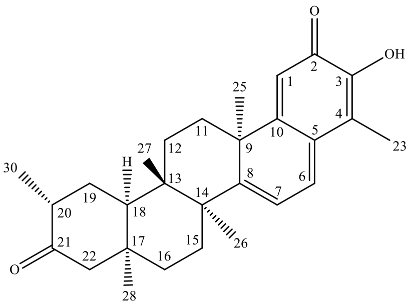

Maytenin

The ^{1}H NMR spectrum of **1** (Figure S1) revealed signals at δ 2.20 (3H); 1.50 (3H); 1.35 (3H); 1.05 (3H); 0.98 (3H) and 0.85 (3H) attributed to 6 methyl groups; a doublet at δ 2.92 associated to H-22; a multiplet at δ 2.50 associated to hydrogen linked to C20; and three signals at δ 7.04 (*d*; 6.7Hz), δ 6.54 (*s*) and δ 6.38 (*d*; 6.7Hz), characteristic of a quinone methide triterpene. The ^{13}CMR spectrum (Figure S2) disclosed signals at δ 41.90, δ 53.53 δ 213.76 assigned to C20, C-22 and C-21, respectively. Comparison of the observed NMR spectra data (Table 1) with values reported in the literature [Rodrigues et al., 2012], together with HSQC and HMBC contour map correlations (Figures S3 and S4) of compound **1** allowed the identification of maytenin (tingenone).

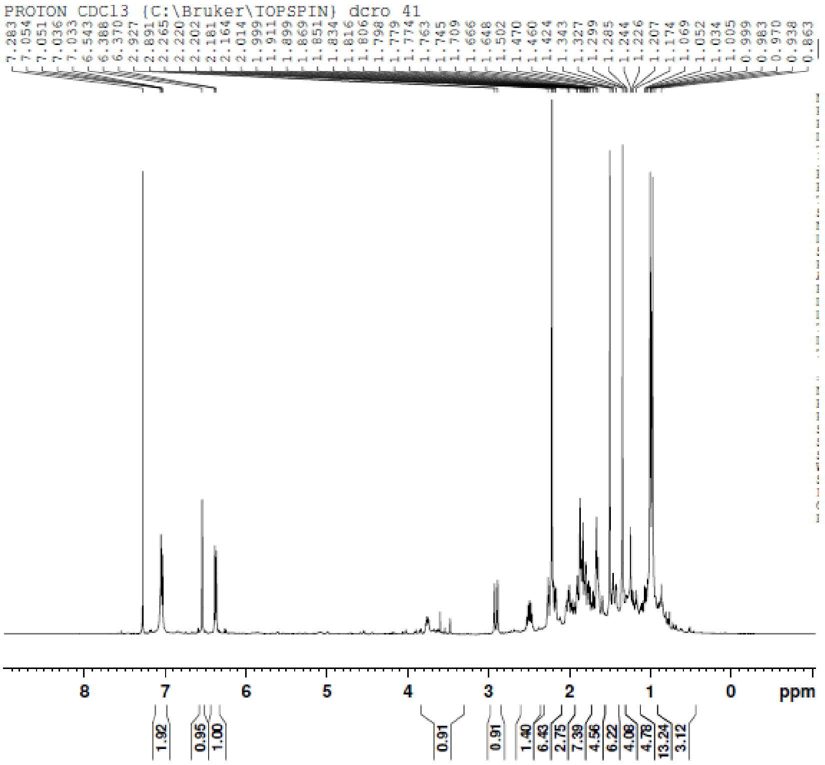

Figure S1: ^{1}H NMR spectrum of maytenin.

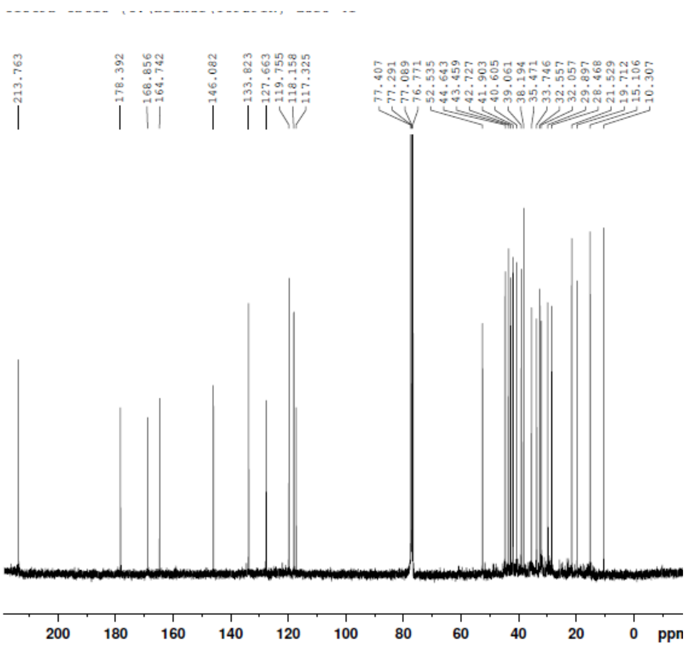

Figure S2: ^{13}CNMR spectrum of maytenin.

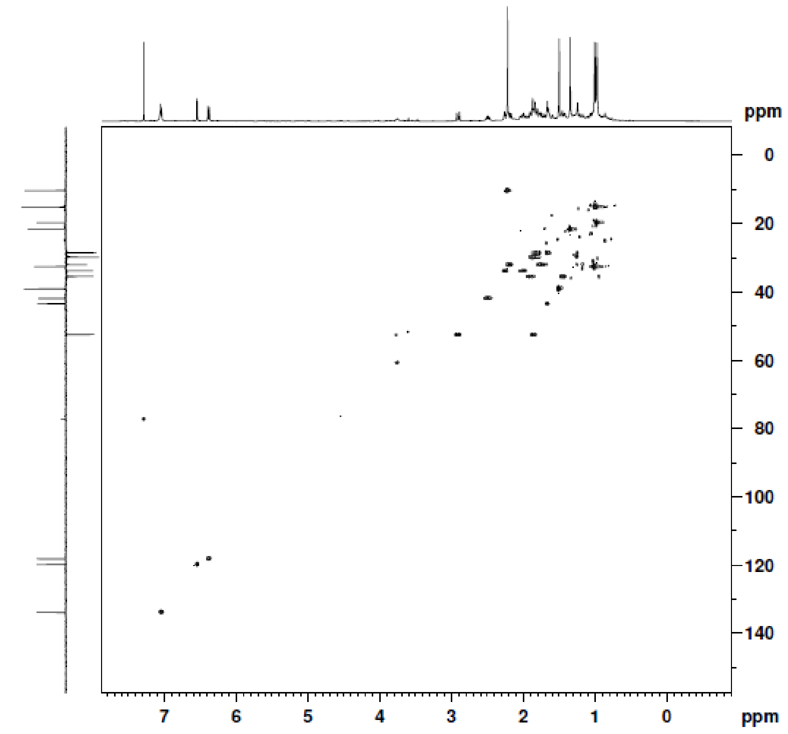

Figure S3: HSQC contour map of maytenin.

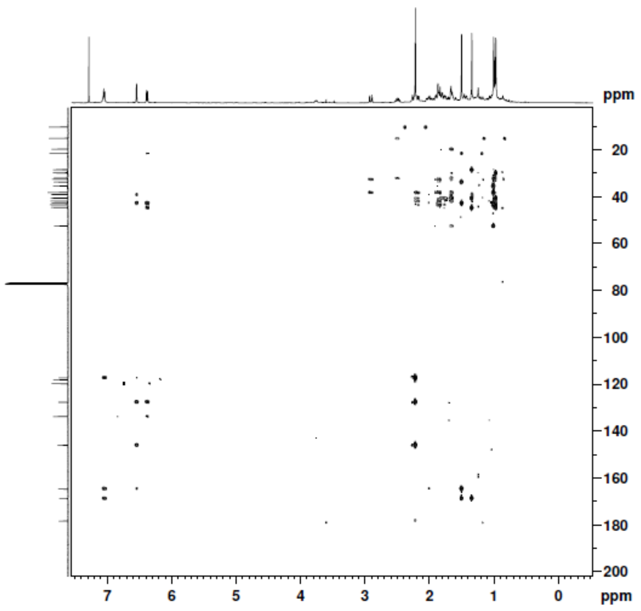

Figure S4: HMBC contour map of maytenin.

Table 1: ^{1}H and ^{13}C NMR data of maitenin in $CDCl_3$.

C/H	^{13}C (δ in ppm)[a]	^{1}H (δ in ppm, *J* in Hz)[b]
**CH**		
1	119.75	6.54 (*s*)
6	133.82	7.04 (*d*; 6.7)
7	118.15	6.38 (*d*; 6.7)
18	43.45	1.59-1.79 (*m*)
20	41.90	2.50 (*m*)
**CH_2**		
11	33.75	2.00 (*m*)<br>2.23 (*m*)
12	29.90	1.85 (*m*)<br>1.83 (*m*)
15	28.47	1.86 (*m*)<br>1.65 (*m*)
16	35.47	1.61 (*m*)<br>2.23 (*m*)
19	32.06	2.20 (*m*)
22	53.53	2.92 (*d*; 14.4)
**C**		
2	178.39	-
3	146.08	-
4	117.32	-
5	127.66	-
8	168.86	-
9	42.73	-
10	164.74	-
13	40.60	-
14	44.64	-
17	38.19	-
21	213.76	-
**OCH_3-23**	10.31	2.20 (*s*)
**OCH_3-25**	39.06	1.50 (*s*)
**OCH_3-26**	21.53	1.35 (*s*)
**OCH_3-27**	19.71	0.98 (*s*)
**OCH_3-28**	32.56	0.85 (*s*)
**OCH_3-30**	15.11	1.05 (*d*; 6.3)

[a]125 MHz. [b]500 MHz. Abbreviations: **d**, doublet; **s**, singlet; **m**, multiplet.

B: 22β- hydroxymaitenin

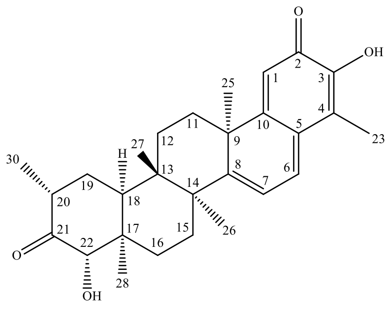

22β- hydroxymaytenin

The ^{1}H NMR spectrum of **2** (Figure S5) revealed three olefinic signals corresponding to H-1, H-6 and H-7, besides methylene hydrogen signals in the region from δ 1.61 to 2.23ppm. It also observed signals at δ 2.21 (3H); 1.50 (3H); 1.35 (3H); 1.05 (3H); 0.96 (3H) and 0.85 (3H) attributed to 6 methyl groups H-23, H-25, H-26, H-30, H-27 e H-28, respectively; the multiplet at δ 2.65 associated to hydrogen linked to C-20, and the singlet at δ 4.53 attributed to H-22. The ^{13}CMR spectrum (Figure S6) disclosed 28 carbon signals, among these, the signals at δ 213.64; δ 76.44 and δ 40.87 assigned to C-21, C-22 and C-20, respectively. From the HSQC and HMBC contour map correlations (Figures S7 and S8) of compound **2** it was possible to assign the signals from the ^{1}H-NMR and ^{13}C-NMR spectra (Table 2). The presence of NOESY correlation between H-22 (δ 4.53) and H-27 (δ 0.96) (Figure S9), and the comparison of the obtained spectral data with results reported in the literature [Likhitwitayawuid et al., 1993] confirmed the structure of 22β-hydroxymaytenin (22β-hydroxytingenone).

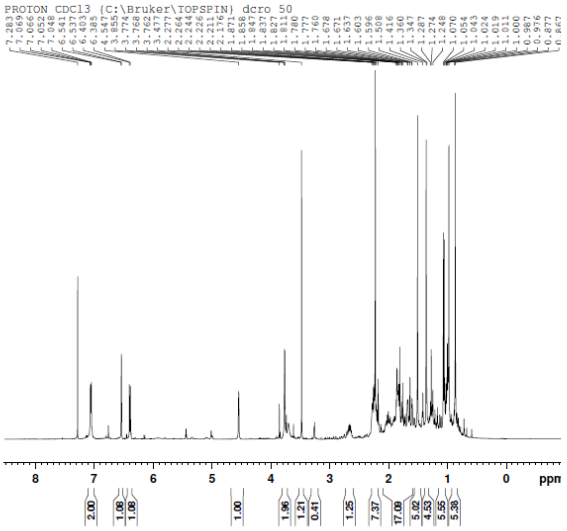

Figure S5: ^{1}H NMR spectrum of 22β-hydroxymaytenin

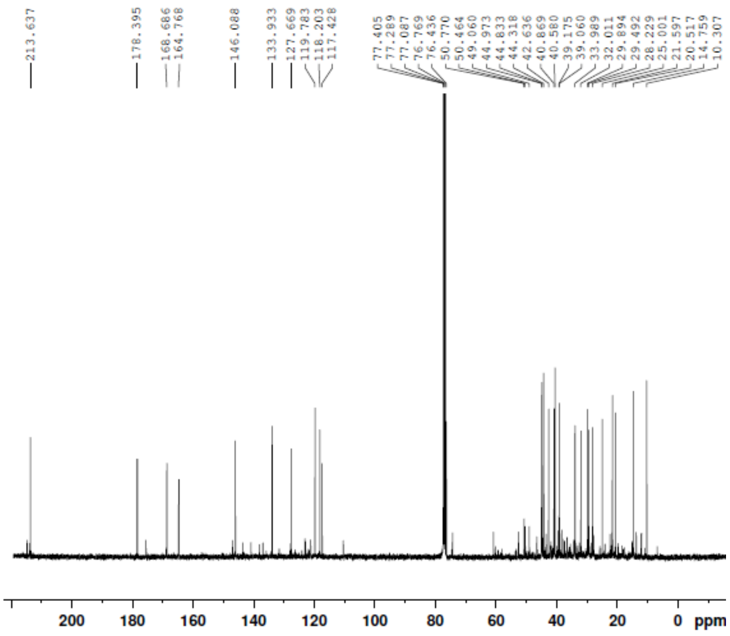

Figure S6: ^{13}C NMR spectrum of 22β-hydroxymaytenin

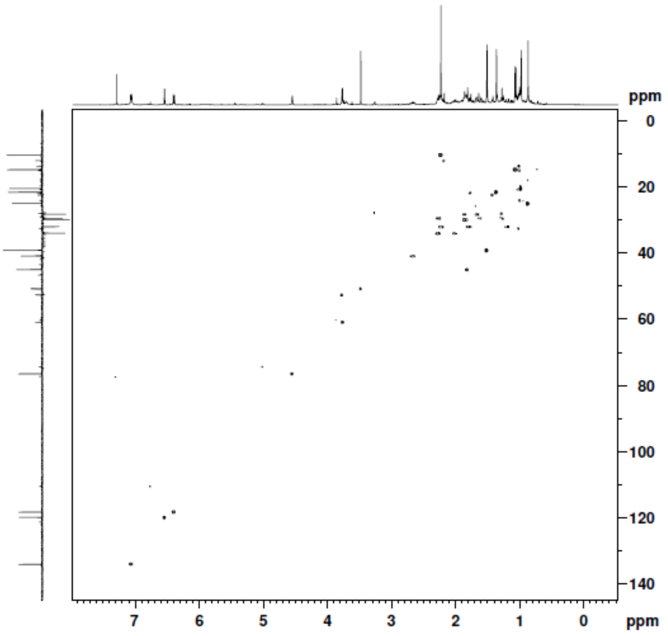

Figure S7: HSQC contour map of 22β-hydroxymaytenin.

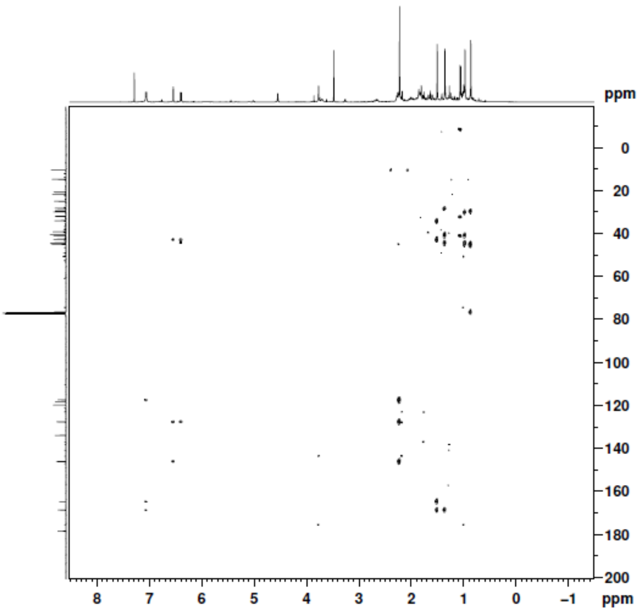

Figure S8: HMQC contour map of 22β-hydroxymaytenin.

Table 1: ^{1}H and ^{13}C NMR data of 22β-hydroxymaitenin in $CDCl_3$.

C/H	^{13}C (δ in ppm)[a]	^{1}H (δ in ppm, *J* in Hz)[b]
**CH**		
1	119.78	6.54 (s)
6	133.93	7.03 (d, 6.7Hz)
7	118.20	6.40 (d, 6.7Hz)
18	44.97	1.80 (m)
20	40.87	2.65 (m)
22	76.44	4.53 (s)
**CH_2**		
11	33.99	2.00 (m)<br>2.23 (m)
12	29.89	1.85 (m)<br>1.83 (m)
15	28.23	1.86 (m)<br>1.65 (m)
16	29.49	1.61 (m)<br>2.23 (m)
19	32.01	2.20 (m)
**C**		
2	178.89	-

3	146.09	-
4	117.43	-
5	127.67	-
8	168.69	-
9	42.63	-
10	164.77	-
13	40.58	-
14	44.32	-
17	44.97	-
21	213.64	-
**OCH_3-23**	10.31	2.21 (3H, s)
**OCH_3-25**	39.17	1.50 (3H, s)
**OCH_3-26**	21.59	1.35 (3H, s)
**OCH_3-27**	20.52	0.96 (3H, s)
**OCH_3-28**	25.00	0.85 (3H, s)
**OCH_3-30**	14.76	1.05 (3H, d, 6.3Hz)

[a]125 MHz. [b]500 MHz. Abbreviations: **d**, doublet; **s**, singlet; **m**, multiplet.

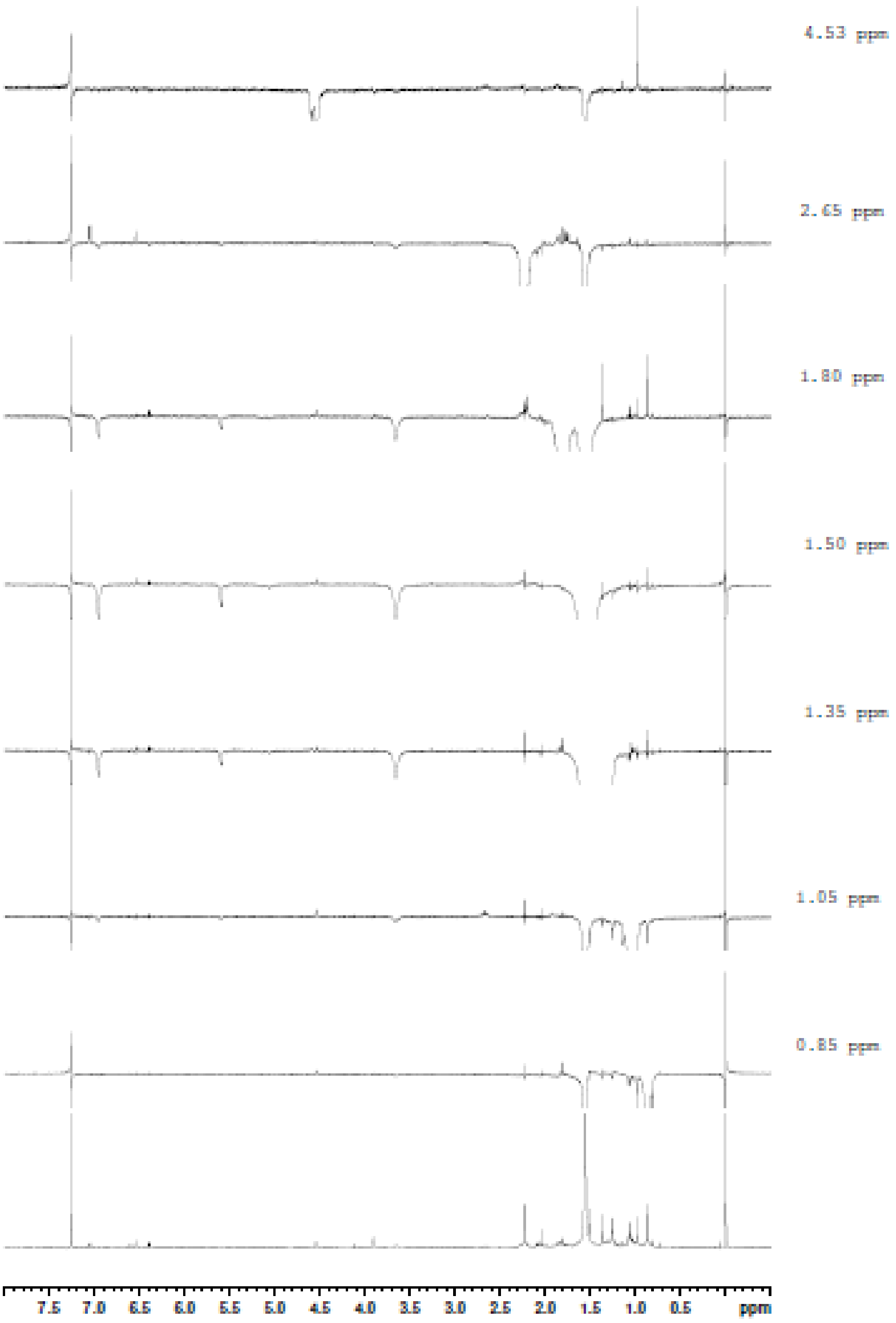

Figure S9: Spectrum for NOESY for 22β-hydroxymaytenin.

References:

Likhitwitayawuid et al., Phytochemistry, Vol. 34, No. 3, pp. 159-763, 1993.

Rodrigues et al., Quim. Nova, Vol. 35, No. 7, 1375-1380, 2012.
