# Supplementary material for: Anticancer Activities of the Quinone-Methide Triterpenes Maytenin and 22-β-hydroxymaytenin Obtained from Cultivated Maytenus ilicifolia Roots Associated with Down-Regulation of miRNA-27a and miR-20a/miR-17-5p
Source: Molecules. 2020 Feb 10;25(3):760. doi: 10.3390/molecules25030760 (PMC7038027; doi:10.3390/molecules25030760)
Supplement: Supplementary file 1 [file molecules-25-00760-s001.zip › Supplementary Table S1 Molecules.pdf]

Supplementary Table S1:
Genomic mutations in the cell lines used in this work, according to the Sanger COSMIC database.

ATCC catalog	Name	Tissue	Histology	Tumor source	Mutant Gene	Mutation
CRL-1628	SCC25	tongue	carcinoma	primary	CDKN2A<br>TP53	c.1_471del471<br>c.625_626delAG
CRL-1629	SCC9	tongue	carcinoma	primary	CDKN2A<br>TP53	c.1_150del150<br>c.822_853del32
HTB-43	FaDu	hypopharynx	carcinoma	primary	CDKN2A<br>SMAD4<br>TP53<br>TP53	c.151-1G>T<br>c.1_1659del1659<br>c.376-1G>A<br>c.743G>T
CCL-138	Detroit 562	pharynx	carcinoma	metastasis	CDKN2A<br>PIK3CA<br>TP53	c.1_457del457<br>c.3140A>G<br>c.524G>A

Source: HEAD AND NECK CANCER PANEL, ATCC (https://www.atcc.org/~/media/5A82DE2BD22A41B2B1D907CBC84A0C3A.ashx)
