# Supplementary material for: Anticancer Activities of the Quinone-Methide Triterpenes Maytenin and 22-β-hydroxymaytenin Obtained from Cultivated Maytenus ilicifolia Roots Associated with Down-Regulation of miRNA-27a and miR-20a/miR-17-5p
Source: Molecules. 2020 Feb 10;25(3):760. doi: 10.3390/molecules25030760 (PMC7038027; doi:10.3390/molecules25030760)
Supplement: Supplementary file 1 [file molecules-25-00760-s001.zip › Supplementary Table S2 Molecules.pdf]

Supplementary Material Table S2

A: Gene expression regulation of miRNAs following treatment of Oral Keratinocytes with maytenin, 22-β-hydroxymaytenin or cisplatin.
FC: Fold-Change Treatment vs Control; K: Keratinocyte.

systematic_ name	FC ([K 22b] vs [K Control])	Regulation ([K 22b] vs [K Control])	FC ([K Maytenin] vs [K Control])	Regulation ([K Maytenin] vs [K Control])	FC ([K Cisplatin] vs [K Control])	Regulation ([K Cisplatin] vs [K Control])	mirbase accession
hsa-let-7a-5p	1.3	up	7.6	up	1.7	up	MIMAT0000062
hsa-let-7b-5p	1.6	up	8.7	up	1.9	up	MIMAT0000063
hsa-let-7c	1.0	up	5.6	up	1.5	up	MIMAT0000064
hsa-let-7d-3p	-1.0	down	28.1	up	16.9	up	MIMAT0004484
hsa-let-7d-5p	20.9	up	102.0	up	-1.0	down	MIMAT0000065
hsa-let-7e-5p	-25.4	down	4.8	up	1.3	up	MIMAT0000066
hsa-let-7f-5p	1.1	up	7.5	up	1.5	up	MIMAT0000067
hsa-let-7g-5p	1.1	up	5.4	up	1.5	up	MIMAT0000414
hsa-let-7i-5p	-1.1	down	4.7	up	1.5	up	MIMAT0000415
hsa-miR-100-5p	26.8	up	240.0	up	36.2	up	MIMAT0000098
hsa-miR-103a-3p	28.5	up	148.6	up	40.6	up	MIMAT0000101
hsa-miR-107	-1.0	down	69.8	up	-1.0	down	MIMAT0000104
hsa-miR-1202	108.5	up	324.1	up	82.8	up	MIMAT0005865
hsa-miR-1207-5p	119.7	up	186.3	up	62.8	up	MIMAT0005871
hsa-miR-1225-5p	157.4	up	218.3	up	74.2	up	MIMAT0005572
hsa-miR-1229-5p	44.2	up	-1.0	down	-1.0	down	MIMAT0022942
hsa-miR-1234-5p	12.4	up	17.9	up	7.9	up	MIMAT0022944
hsa-miR-1246	35.5	up	112.1	up	-1.0	down	MIMAT0005898
hsa-miR-125a-5p	-1.0	down	84.1	up	-1.0	down	MIMAT0000443
hsa-miR-125b-5p	-2.3	down	3.6	up	1.1	up	MIMAT0000423
hsa-miR-1260a	1.4	up	3.7	up	1.8	up	MIMAT0005911

systematic_ name	FC ([K 22b] vs [K Control])	Regulation ([K 22b] vs [K Control])	FC ([K Maytenin] vs [K Control])	Regulation ([K Maytenin] vs [K Control])	FC ([K Cisplatin] vs [K Control])	Regulation ([K Cisplatin] vs [K Control])	mirbase accession
hsa-miR-1260b	1.2	up	5.1	up	1.9	up	MIMAT0015041
hsa-miR-1268a	82.9	up	83.6	up	32.4	up	MIMAT0005922
hsa-miR-1273g-3p	1.7	up	6.7	up	1.9	up	MIMAT0022742
hsa-miR-130a-3p	1.1	up	7.3	up	1.6	up	MIMAT0000425
hsa-miR-135a-5p	-1.0	down	75.6	up	-1.0	down	MIMAT0000428
hsa-miR-135b-5p	20.8	up	156.6	up	-1.0	down	MIMAT0000758
hsa-miR-141-3p	2.7	up	7.3	up	3.8	up	MIMAT0000432
hsa-miR-15b-5p	-1.0	down	61.2	up	-1.0	down	MIMAT0000417
hsa-miR-16-5p	1.4	up	5.3	up	1.7	up	MIMAT0000069
hsa-miR-17-5p	-1.0	down	55.4	up	-1.0	down	MIMAT0000070
hsa-miR-188-5p	51.9	up	-1.0	down	-1.0	down	MIMAT0000457
hsa-miR-191-3p	-1.1	down	-11.4	down	-11.4	down	MIMAT0001618
hsa-miR-1915-3p	174.1	up	170.6	up	95.3	up	MIMAT0007892
hsa-miR-193b-3p	22.4	up	142.2	up	40.0	up	MIMAT0002819
hsa-miR-196a-5p	-1.0	down	59.2	up	-1.0	down	MIMAT0000226
hsa-miR-196b-5p	-1.0	down	70.9	up	-1.0	down	MIMAT0001080
hsa-miR-199a-3p	-40.1	down	4.3	up	-1.1	down	MIMAT0000232
hsa-miR-19b-3p	1.4	up	6.1	up	2.1	up	MIMAT0000074
hsa-miR-200b-3p	36.3	up	189.3	up	42.6	up	MIMAT0000318
hsa-miR-200c-3p	2.1	up	11.7	up	3.5	up	MIMAT0000617
hsa-miR-203a	1.8	up	14.0	up	2.5	up	MIMAT0000264
hsa-miR-204-5p	-1.0	down	60.0	up	-1.0	down	MIMAT0000265
hsa-miR-205-5p	1.5	up	8.2	up	2.4	up	MIMAT0000266
hsa-miR-20a-5p	1.7	up	8.3	up	3.4	up	MIMAT0000075
hsa-miR-21-5p	1.4	up	5.7	up	2.1	up	MIMAT0000076
hsa-miR-210	-1.0	down	59.9	up	-1.0	down	MIMAT0000267

systematic_ name	FC ([K 22b] vs [K Control])	Regulation ([K 22b] vs [K Control])	FC ([K Maytenin] vs [K Control])	Regulation ([K Maytenin] vs [K Control])	FC ([K Cisplatin] vs [K Control])	Regulation ([K Cisplatin] vs [K Control])	mirbase accession
hsa-miR-211-5p	-1.0	down	142.2	up	-1.0	down	MIMAT0000268
hsa-miR-22-3p	1.3	up	5.9	up	1.8	up	MIMAT0000077
hsa-miR-222-3p	-1.0	down	81.4	up	-1.0	down	MIMAT0000279
hsa-miR-23a-3p	1.5	up	11.1	up	2.1	up	MIMAT0000078
hsa-miR-23b-3p	-1.0	down	62.0	up	-1.0	down	MIMAT0000418
hsa-miR-24-3p	1.5	up	7.5	up	2.1	up	MIMAT0000080
hsa-miR-26a-2-3p	-1.0	down	27.1	up	-1.0	down	MIMAT0004681
hsa-miR-27a-3p	1.9	up	7.9	up	2.9	up	MIMAT0000084
hsa-miR-27b-3p	-1.0	down	72.4	up	-1.0	down	MIMAT0000419
hsa-miR-2861	196.3	up	160.0	up	62.2	up	MIMAT0013802
hsa-miR-29a-3p	1.2	up	6.2	up	1.6	up	MIMAT0000086
hsa-miR-29a-5p	-1.0	down	95.7	up	-1.0	down	MIMAT0004503
hsa-miR-29b-3p	1.6	up	4.7	up	2.1	up	MIMAT0000100
hsa-miR-29c-3p	-1.0	down	64.2	up	-1.0	down	MIMAT0000681
hsa-miR-302a-3p	-1.0	down	65.8	up	-1.0	down	MIMAT0000684
hsa-miR-302b-3p	-1.0	down	31.6	up	-1.0	down	MIMAT0000715
hsa-miR-31-3p	-1.0	down	61.3	up	-1.0	down	MIMAT0004504
hsa-miR-31-5p	-1.0	down	103.9	up	34.4	up	MIMAT0000089
hsa-miR-3121-5p	-1.0	down	58.4	up	-1.0	down	MIMAT0019199
hsa-miR-3162-5p	177.5	up	368.2	up	111.0	up	MIMAT0015036
hsa-miR-3173-5p	-1.0	down	71.1	up	-1.0	down	MIMAT0019214
hsa-miR-3195	57.4	up	65.7	up	33.1	up	MIMAT0015079
hsa-miR-3196	43.1	up	-1.0	down	-1.0	down	MIMAT0015080
hsa-miR-320b	-1.0	down	57.7	up	-1.0	down	MIMAT0005792
hsa-miR-328	-1.0	down	130.0	up	34.5	up	MIMAT0000752
hsa-miR-335-3p	-1.0	down	59.3	up	-1.0	down	MIMAT0004703

systematic_ name	FC ([K 22b] vs [K Control])	Regulation ([K 22b] vs [K Control])	FC ([K Maytenin] vs [K Control])	Regulation ([K Maytenin] vs [K Control])	FC ([K Cisplatin] vs [K Control])	Regulation ([K Cisplatin] vs [K Control])	mirbase accession
hsa-miR-337-3p	-1.0	down	149.5	up	-1.0	down	MIMAT0000754
hsa-miR-34a-5p	1.2	up	7.6	up	1.7	up	MIMAT0000255
hsa-miR-3613-5p	-1.0	down	62.2	up	-1.0	down	MIMAT0017990
hsa-miR-3617-3p	-1.0	down	146.5	up	-1.0	down	MIMAT0022966
hsa-miR-3656	4.6	up	3.3	up	3.8	up	MIMAT0018076
hsa-miR-3665	152.5	up	186.9	up	52.4	up	MIMAT0018087
hsa-miR-3676-5p	9.4	up	18.6	up	7.9	up	MIMAT0022734
hsa-miR-3679-5p	22.3	up	-1.0	down	-1.0	down	MIMAT0018104
hsa-miR-376b-5p	-1.0	down	59.2	up	-1.0	down	MIMAT0022923
hsa-miR-376c-5p	-1.0	down	59.6	up	-1.0	down	MIMAT0022861
hsa-miR-3940-5p	33.0	up	-1.0	down	-1.0	down	MIMAT0019229
hsa-miR-3960	7.3	up	9.3	up	4.3	up	MIMAT0019337
hsa-miR-424-5p	-1.0	down	71.0	up	-1.0	down	MIMAT0001341
hsa-miR-4281	5.5	up	8.5	up	3.8	up	MIMAT0016907
hsa-miR-4284	1.2	up	6.2	up	1.4	up	MIMAT0016915
hsa-miR-4286	2.0	up	5.8	up	2.4	up	MIMAT0016916
hsa-miR-4290	-1.0	down	59.3	up	-1.0	down	MIMAT0016921
hsa-miR-4299	49.2	up	-1.0	down	-1.0	down	MIMAT0016851
hsa-miR-4307	-1.0	down	134.2	up	-1.0	down	MIMAT0016860
hsa-miR-4443	95.8	up	115.7	up	48.6	up	MIMAT0018961
hsa-miR-4454	1.4	up	4.1	up	1.9	up	MIMAT0018976
hsa-miR-4455	-1.0	down	57.9	up	-1.0	down	MIMAT0018977
hsa-miR-4459	16.6	up	31.5	up	7.0	up	MIMAT0018981
hsa-miR-4466	122.2	up	136.4	up	83.1	up	MIMAT0018993
hsa-miR-4505	1.2	up	2.3	up	1.2	up	MIMAT0019041
hsa-miR-4516	13.5	up	16.4	up	3.4	up	MIMAT0019053

systematic_ name	FC ([K 22b] vs [K Control])	Regulation ([K 22b] vs [K Control])	FC ([K Maytenin] vs [K Control])	Regulation ([K Maytenin] vs [K Control])	FC ([K Cisplatin] vs [K Control])	Regulation ([K Cisplatin] vs [K Control])	mirbase accession
hsa-miR-4530	228.4	up	427.5	up	110.0	up	MIMAT0019069
hsa-miR-4665-3p	1.6	up	-47.0	down	1.3	up	MIMAT0019740
hsa-miR-4668-3p	-1.0	down	65.6	up	-1.0	down	MIMAT0019746
hsa-miR-4687-3p	189.1	up	268.7	up	56.4	up	MIMAT0019775
hsa-miR-4694-5p	-1.0	down	63.5	up	-1.0	down	MIMAT0019786
hsa-miR-4739	29.7	up	-1.0	down	-1.0	down	MIMAT0019868
hsa-miR-4741	24.4	up	-1.0	down	-1.0	down	MIMAT0019871
hsa-miR-4753-3p	-1.0	down	57.8	up	-1.0	down	MIMAT0019891
hsa-miR-4763-3p	53.8	up	-1.0	down	-1.0	down	MIMAT0019913
hsa-miR-4787-5p	44.8	up	61.4	up	-1.0	down	MIMAT0019956
hsa-miR-483-3p	-1.0	down	120.2	up	-1.0	down	MIMAT0002173
hsa-miR-494	29.6	up	97.5	up	154.2	up	MIMAT0002816
hsa-miR-5001-5p	44.5	up	-1.0	down	-1.0	down	MIMAT0021021
hsa-miR-5100	1.5	up	5.2	up	2.2	up	MIMAT0022259
hsa-miR-519e-3p	-1.0	down	63.7	up	-1.0	down	MIMAT0002829
hsa-miR-520d-3p	-1.0	down	64.6	up	-1.0	down	MIMAT0002856
hsa-miR-548ag	-1.0	down	57.3	up	-1.0	down	MIMAT0018969
hsa-miR-548aq-5p	-1.0	down	38.8	up	-1.0	down	MIMAT0022263
hsa-miR-548az-5p	-1.0	down	62.5	up	-1.0	down	MIMAT0025456
hsa-miR-548d-5p	-1.0	down	126.9	up	-1.0	down	MIMAT0004812
hsa-miR-548m	-1.0	down	124.7	up	-1.0	down	MIMAT0005917
hsa-miR-5703	78.7	up	116.9	up	-1.0	down	MIMAT0022496
hsa-miR-572	34.0	up	-1.0	down	-1.0	down	MIMAT0003237
hsa-miR-5739	1.8	up	7.1	up	2.4	up	MIMAT0023116
hsa-miR-574-5p	23.1	up	156.4	up	-1.0	down	MIMAT0004795
hsa-miR-576-5p	-1.0	down	60.1	up	-1.0	down	MIMAT0003241

systematic_ name	FC ([K 22b] vs [K Control])	Regulation ([K 22b] vs [K Control])	FC ([K Maytenin] vs [K Control])	Regulation ([K Maytenin] vs [K Control])	FC ([K Cisplatin] vs [K Control])	Regulation ([K Cisplatin] vs [K Control])	mirbase accession
hsa-miR-5787	226.0	up	363.3	up	31.1	up	MIMAT0023252
hsa-miR-6068	71.8	up	-1.0	down	-1.0	down	MIMAT0023693
hsa-miR-6085	1.5	up	7.1	up	2.2	up	MIMAT0023710
hsa-miR-6087	8.4	up	10.7	up	2.9	up	MIMAT0023712
hsa-miR-6088	8.7	up	10.2	up	6.5	up	MIMAT0023713
hsa-miR-6089	5.7	up	7.0	up	3.3	up	MIMAT0023714
hsa-miR-6090	8.7	up	9.5	up	4.3	up	MIMAT0023715
hsa-miR-6125	6.7	up	8.1	up	2.8	up	MIMAT0024598
hsa-miR-6126	24.3	up	-1.0	down	-1.0	down	MIMAT0024599
hsa-miR-613	-1.0	down	68.3	up	-1.0	down	MIMAT0003281
hsa-miR-6165	63.8	up	260.5	up	92.0	up	MIMAT0024782
hsa-miR-630	106.5	up	183.8	up	-1.0	down	MIMAT0003299
hsa-miR-638	13.8	up	15.7	up	5.4	up	MIMAT0003308
hsa-miR-642a-3p	95.0	up	250.6	up	-1.0	down	MIMAT0020924
hsa-miR-642b-3p	41.9	up	-1.0	down	-1.0	down	MIMAT0018444
hsa-miR-6507-3p	-1.0	down	150.9	up	-1.0	down	MIMAT0025471
hsa-miR-6724-5p	45.5	up	-1.0	down	-1.0	down	MIMAT0025856
hsa-miR-762	63.2	up	58.8	up	-1.0	down	MIMAT0010313
hsa-miR-940	2.2	up	-23.4	down	1.5	up	MIMAT0004983
hsa-miR-96-5p	-1.0	down	59.2	up	-1.0	down	MIMAT0000095

B: Gene expression regulation of miRNAs following treatment of SCC25 cell line with maytenin, 22-β-hydroxymaytenin or cisplatin.
FC: Fold-Change Treatment vs Control.

systematic_ name	FC ([SCC25 22b] vs [SCC25 Control])	Regulation ([SCC25 22b] vs [SCC25 Control])	FC ([SCC25 Maytenin] vs [SCC25 Control])	Regulation ([SCC25 Maytenin] vs [SCC25 Control])	FC ([SCC25 Cisplatin] vs [SCC25 Control])	Regulation ([SCC25 Cisplatin] vs [SCC25 Control])	mirbase accession
hsa-let-7a-5p	-186.1	down	-186.1	down	1.0	up	MIMAT0000062
hsa-let-7b-5p	-125.8	down	-125.8	down	1.1	up	MIMAT0000063
hsa-let-7c	-1.0	down	-1.0	down	21.9	up	MIMAT0000064
hsa-let-7d-5p	-46.2	down	-46.2	down	-1.1	down	MIMAT0000065
hsa-let-7e-5p	-1.0	down	-1.0	down	22.9	up	MIMAT0000066
hsa-let-7f-5p	-84.0	down	-84.0	down	1.1	up	MIMAT0000067
hsa-let-7g-5p	-1.0	down	-1.0	down	21.9	up	MIMAT0000414
hsa-let-7i-5p	-62.0	down	-62.0	down	1.0	up	MIMAT0000415
hsa-miR-100-5p	-44.0	down	-44.0	down	-1.2	down	MIMAT0000098
hsa-miR-103a-3p	-40.5	down	-40.5	down	1.0	up	MIMAT0000101
hsa-miR-1234-5p	-22.5	down	-22.5	down	1.1	up	MIMAT0022944
hsa-miR-1238-3p	-1.0	down	-1.0	down	10.4	up	MIMAT0005593
hsa-miR-1246	-29.1	down	-29.1	down	4.5	up	MIMAT0005898
hsa-miR-1260a	-337.3	down	-337.3	down	1.3	up	MIMAT0005911
hsa-miR-1260b	-411.2	down	-411.2	down	1.0	up	MIMAT0015041

systematic_ name	FC ([SCC25 22b] vs [SCC25 Control])	Regulation ([SCC25 22b] vs [SCC25 Control])	FC ([SCC25 Maytenin] vs [SCC25 Control])	Regulation ([SCC25 Maytenin] vs [SCC25 Control])	FC ([SCC25 Cisplatin] vs [SCC25 Control])	Regulation ([SCC25 Cisplatin] vs [SCC25 Control])	mirbase accession
hsa-miR-1268a	-1.0	down	-1.0	down	25.1	up	MIMAT0005922
hsa-miR-1273g-3p	-671.7	down	-20.2	down	1.2	up	MIMAT0022742
hsa-miR-1290	-1.0	down	-1.0	down	27.0	up	MIMAT0005880
hsa-miR-141-3p	-45.0	down	-45.0	down	-1.0	down	MIMAT0000432
hsa-miR-15b-5p	-92.7	down	-92.7	down	-1.1	down	MIMAT0000417
hsa-miR-16-5p	-88.4	down	-88.4	down	1.3	up	MIMAT0000069
hsa-miR-17-5p	-36.4	down	-36.4	down	-1.3	down	MIMAT0000070
hsa-miR-191-3p	-24.8	down	-24.8	down	-24.8	down	MIMAT0001618
hsa-miR-193b-3p	-23.4	down	-23.4	down	-23.4	down	MIMAT0002819
hsa-miR-19b-3p	-45.6	down	-45.6	down	-1.4	down	MIMAT0000074
hsa-miR-200b-3p	-28.0	down	-28.0	down	-1.0	down	MIMAT0000318
hsa-miR-200c-3p	-36.9	down	-36.9	down	1.1	up	MIMAT0000617
hsa-miR-205-5p	-433.6	down	-433.6	down	-1.3	down	MIMAT0000266
hsa-miR-20a-5p	-88.1	down	-88.1	down	-1.1	down	MIMAT0000075
hsa-miR-21-5p	-524.3	down	-524.3	down	1.0	up	MIMAT0000076
hsa-miR-22-3p	-23.0	down	-23.0	down	1.7	up	MIMAT0000077

systematic_name	FC ([SCC25 22b] vs [SCC25 Control])	Regulation ([SCC25 22b] vs [SCC25 Control])	FC ([SCC25 Maytenin] vs [SCC25 Control])	Regulation ([SCC25 Maytenin] vs [SCC25 Control])	FC ([SCC25 Cisplatin] vs [SCC25 Control])	Regulation ([SCC25 Cisplatin] vs [SCC25 Control])	mirbase accession
hsa-miR-23a-3p	-35.6	down	-35.6	down	1.4	up	MIMAT0000078
hsa-miR-23b-3p	-32.7	down	-32.7	down	1.1	up	MIMAT0000418
hsa-miR-24-3p	-157.2	down	-157.2	down	-1.0	down	MIMAT0000080
hsa-miR-27a-3p	-62.0	down	-62.0	down	-1.2	down	MIMAT0000084
hsa-miR-27b-3p	-36.6	down	-36.6	down	-1.1	down	MIMAT0000419
hsa-miR-2861	-37.3	down	-37.3	down	-1.1	down	MIMAT0013802
hsa-miR-29a-3p	-64.3	down	-64.3	down	-1.0	down	MIMAT0000086
hsa-miR-29b-3p	-27.3	down	-27.3	down	-1.3	down	MIMAT0000100
hsa-miR-3162-5p	-50.9	down	-50.9	down	-50.9	down	MIMAT0015036
hsa-miR-3651	-28.8	down	-28.8	down	-1.3	down	MIMAT0018071
hsa-miR-3656	-30.1	down	-30.1	down	-1.4	down	MIMAT0018076
hsa-miR-3665	-1.0	down	-1.0	down	21.7	up	MIMAT0018087
hsa-miR-3676-5p	-1.0	down	-1.0	down	33.6	up	MIMAT0022734
hsa-miR-3960	-57.2	down	-57.2	down	1.5	up	MIMAT0019337
hsa-miR-4281	-32.5	down	-32.5	down	1.3	up	MIMAT0016907
hsa-miR-4284	-58.9	down	-58.9	down	-1.2	down	MIMAT0016915

systematic_ name	FC ([SCC25 22b] vs [SCC25 Control])	Regulation ([SCC25 22b] vs [SCC25 Control])	FC ([SCC25 Maytenin] vs [SCC25 Control])	Regulation ([SCC25 Maytenin] vs [SCC25 Control])	FC ([SCC25 Cisplatin] vs [SCC25 Control])	Regulation ([SCC25 Cisplatin] vs [SCC25 Control])	mirbase accession
hsa-miR-4286	-181.4	down	-181.4	down	1.0	up	MIMAT0016916
hsa-miR-4433-5p	-1.0	down	23.0	up	19.8	up	MIMAT0020956
hsa-miR-4454	-218.1	down	-20.4	down	-1.1	down	MIMAT0018976
hsa-miR-4459	-155.2	down	-155.2	down	1.8	up	MIMAT0018981
hsa-miR-4505	-86.1	down	-86.1	down	-2.2	down	MIMAT0019041
hsa-miR-4507	-49.9	down	-49.9	down	-49.9	down	MIMAT0019044
hsa-miR-4516	-100.7	down	-100.7	down	1.4	up	MIMAT0019053
hsa-miR-4787-5p	-24.5	down	-24.5	down	-1.2	down	MIMAT0019956
hsa-miR-494	-36.7	down	-36.7	down	-36.7	down	MIMAT0002816
hsa-miR-5100	-657.4	down	-21.4	down	1.1	up	MIMAT0022259
hsa-miR-548b-5p	-1.0	down	-1.0	down	10.0	up	MIMAT0004798
hsa-miR-5739	-70.5	down	-70.5	down	-1.2	down	MIMAT0023116
hsa-miR-6069	-23.7	down	-23.7	down	-23.7	down	MIMAT0023694
hsa-miR-6085	-58.0	down	-58.0	down	-2.9	down	MIMAT0023710
hsa-miR-6087	-194.0	down	-194.0	down	-1.3	down	MIMAT0023712
hsa-miR-6088	-25.9	down	-25.9	down	-25.9	down	MIMAT0023713

systematic_ name	FC ([SCC25 22b] vs [SCC25 Control])	Regulation ([SCC25 22b] vs [SCC25 Control])	FC ([SCC25 Maytenin] vs [SCC25 Control])	Regulation ([SCC25 Maytenin] vs [SCC25 Control])	FC ([SCC25 Cisplatin] vs [SCC25 Control])	Regulation ([SCC25 Cisplatin] vs [SCC25 Control])	mirbase accession
hsa-miR-6089	-480.1	down	-6.7	down	1.1	up	MIMAT0023714
hsa-miR-6090	-27.0	down	-27.0	down	4.1	up	MIMAT0023715
hsa-miR-6125	-108.5	down	-108.5	down	-1.2	down	MIMAT0024598
hsa-miR-638	-43.6	down	-43.6	down	1.0	up	MIMAT0003308
hsa-miR-6508-5p	-1.0	down	21.7	up	-1.0	down	MIMAT0025472
hsa-miR-940	-24.0	down	-1.1	down	-1.0	down	MIMAT0004983
